# A catalog of mRNAs transferred from males to females during mating in *Drosophila arizonae* and *D. mojavensis*

**DOI:** 10.1371/journal.pone.0358942

**Published:** 2026-10-08

**Authors:** Juan Hurtado, Carson W. Allan, Sergio Castrezana, Jeremy M. Bono, Luciano M. Matzkin

**Affiliations:** 1 Department of Entomology, University of Arizona, Tucson, Arizona, United States of America; 2 Department of Biology, University of Colorado Colorado Springs, Colorado Springs, Colorado, United States of America; 3 BIO5 Institute, University of Arizona, Tucson, Arizona, United States of America; 4 Department of Ecology and Evolutionary Biology, Tucson, Arizona, United States of America; Universite Clermont Auvergne, FRANCE

## Abstract

mRNA is a common component of male ejaculates across diverse organisms, yet whether these male-derived mRNAs (mdRNAs) serve biological functions in females or represent incidental byproducts of ejaculate composition remains unresolved. Recent work in *Drosophilq arizonae* demonstrated that mdRNAs are translated by female reproductive tract cells into proteins, and that genes encoding them have functional postmating effects, suggesting that at least some mdRNAs are biologically important. A systematic characterization of the mdRNA repertoire in this species is therefore a necessary next step. Here we present a catalog of mdRNAs in *D. arizonae* and its sister species *D. mojavensis*, identified through RNA-seq profiling of the female reproductive tract following heterospecific crosses, with expression data from male reproductive tract organs to classify candidates by tissue of origin. We identified 270 candidate mdRNAs whose abundance in the reproductive tract of mated females correlates with expression in testes, sperm-filled seminal vesicles, and accessory glands, consistent with multiple tissues contributing to the pool of mdRNAs. Functional enrichment analyses reveal overrepresentation of ribosomal proteins, suggesting potential roles in translational regulation within the female reproductive tract. To investigate how sperm-associated mdRNAs might become available for translation in the female reproductive tract, we generated a GFP-tagged sperm line and tracked sperm fate following mating. A substantial proportion of transferred sperm became trapped within the insemination reaction mass that forms in the uterus during copulation, possibly releasing RNA into the female reproductive environment. This catalog provides a foundation for future studies on the biological significance of mdRNAs.

## Introduction

During mating, males transfer to females a complex mixture of ejaculate components that profoundly influence female physiology and reproductive success [1–3]. These components include both sperm cells and the seminal fluid that accompanies them, each potentially contributing distinct molecular signals to the female reproductive environment. Among seminal components, seminal fluid proteins have been extensively studied, particularly in *Drosophila melanogaster*, where they modulate female receptivity, egg-laying rate, and immune responses [4–7].

More recently, attention has expanded beyond proteins to other classes of ejaculate molecules, and RNA has emerged as a widespread component of male ejaculates across diverse organisms, including insects and mammals [8–13]. Sperm themselves carry transcripts into the female reproductive tract, and paternal mRNAs delivered to the oocyte at fertilization can shape early embryonic gene expression [14,15]. In mammals, sperm-associated RNA can further influence offspring phenotypes, including metabolic traits that track paternal diet [16–18]. Whether sperm-associated mRNAs are only carried within the sperm cell or associated with vesicles adhering to the sperm surface remains unclear: in mammals, extracellular vesicles from the epididymal epithelium fuse with or adhere to maturing sperm and can remodel their RNA content [19], while in *Drosophila*, exosomes secreted by secondary cells of the male accessory glands have been shown to bind to sperm [20]. Seminal fluid also carries RNA independently of sperm, packaged within extracellular vesicles secreted by the epididymal epithelium (mammals) [9,21,22] and probably by the male accessory glands (insects) [10,11]. Despite the recent progress, the functional significance of male-derived RNA transferred to females, particularly mRNA, remains largely unresolved.

The first evidence of mRNA transfer during insect mating came from a study in *D. mojavensis* and *D. arizonae*, where Bono et al. [8] used microarray profiling of the female lower reproductive tract following heterospecific crosses to demonstrate that at least seven transcripts detected in mated females were of male origin. This discovery raised the question of whether transferred RNAs serve functional roles in the female or represent incidental components of the ejaculate. Direct evidence of their role was provided in Matzkin et al. [13], who demonstrated in *D. arizonae* that at least some male-derived mRNAs (hereafter mdRNAs) are translated into protein by female reproductive tract cells, producing male-derived female-translated proteins (mdFTPs), and that at least some of the genes encoding mdFTPs have functional effects on postmating phenotypes. Matzkin et al. [13] identified mdFTPs using SILAC-based mass spectrometry to characterize the proteome of heavy-labeled *D. mojavensis* female reproductive tracts inseminated by unlabeled *D. arizonae* males. This approach allowed mdFTPs to be distinguished from female-derived proteins or seminal fluid proteins based on species-specific peptide sequences and the presence of heavy-labeled lysine. These findings strongly suggest that mdRNAs are not merely incidental ejaculate components but can carry biological information that is functionally decoded in female tissues.

Understanding the biological significance of mdRNA transfer requires establishing a comprehensive catalog of these molecules and identifying their tissue of origin within the male reproductive tract. In *Drosophila*, the male reproductive tract includes several secretory compartments (Fig 1A): the testes, which produce sperm; the male accessory glands, which synthesize the majority of seminal fluid proteins; and the ejaculatory duct and bulb, which contribute additional secretory products to the ejaculate [5]. Each of these compartments represents a potential source of mdRNAs.

**Fig 1:**
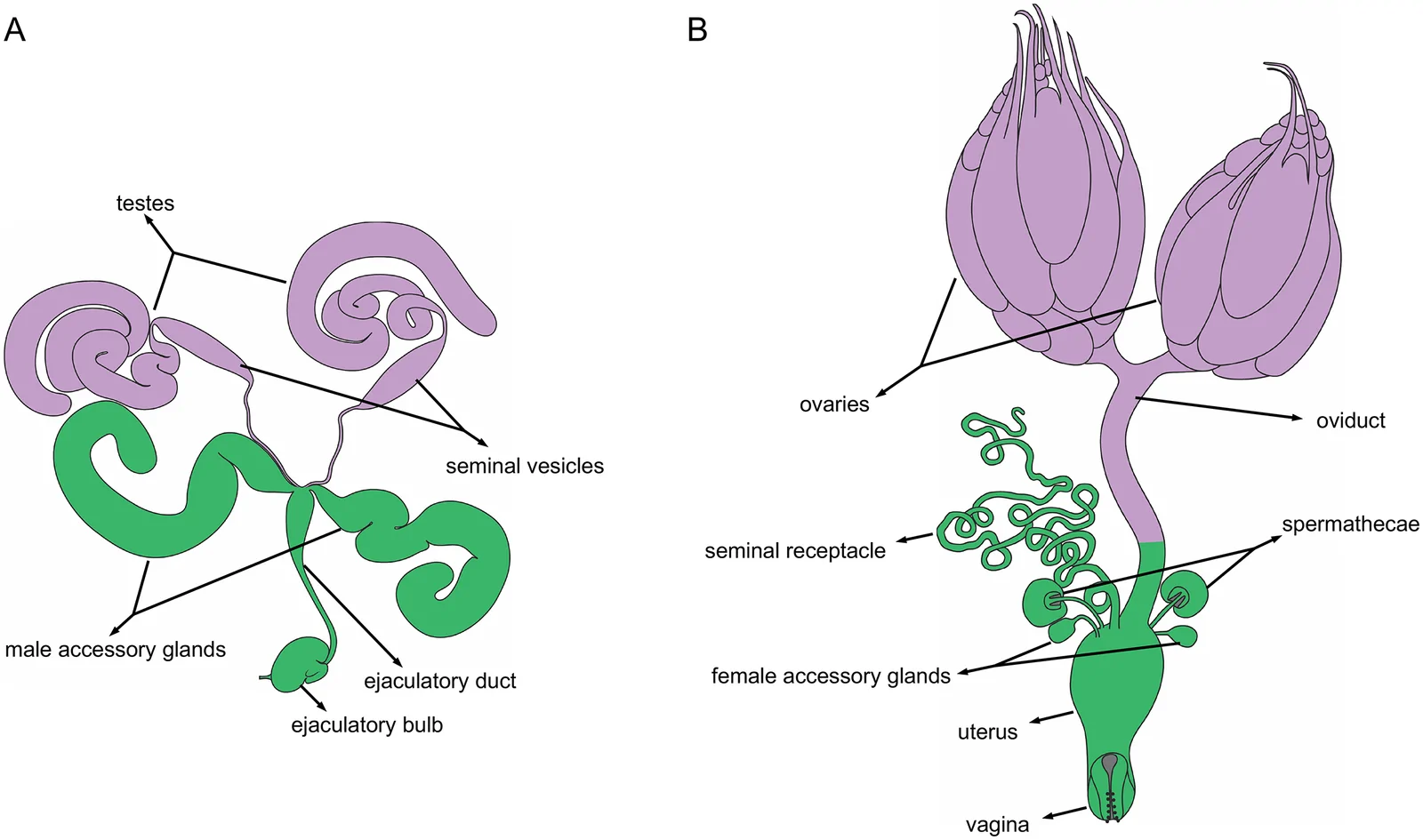
Schematic of the *D. arizonae* male and female reproductive tracts highlighting the compartments relevant to mdRNA transfer. (A) Male reproductive tract, showing the testes, including the seminal vesicles (SVs), in purple, and the male accessory glands, the ejaculatory bulb, and the ejaculatory duct (collectively referred to as MAGs throughout this study) in green. (B) Female reproductive tract, showing ovaries and oviduct in purple, and the lower reproductive tract (LRT), including the uterus, the seminal receptacle, the spermathecae, the female accessory glands, and the vagina, in green.

While transportation of intact mdRNAs within sperm is well-established in insects and mammals [12,14,23], their transfer via seminal exosomes or other extracellular vesicles has yet to be confirmed in insects. As exosomes are known to protect RNA from degradation and facilitate intercellular RNA transfer [24], they provide a mechanism by which mdRNAs encoding mdFTPs could be internalized by recipient cells of the female reproductive tract. In *D. melanogaster*, in fact, exosomes secreted by the secondary cells of the male accessory gland interact with epithelial cells of the female reproductive tract [20]. However, Cridland et al. [12] found that the majority of mdRNAs in *D. melanogaster* show expression consistent with a sperm origin, suggesting that mdRNAs may be primarily carried within sperm cells rather than secreted by accessory glands into the seminal fluid.

Should some mdFTP-coding mdRNAs be carried within sperm cells rather than exosomes, an important mechanistic question arises: how could they become accessible to female reproductive tract cells for translation? The plasma membrane of intact sperm would represent a barrier to RNA release, and the mechanisms by which this barrier might be overcome remain unclear. Moreover, given that mdFTPs are detectable shortly after copulation [13], any viable mechanism must operate rapidly, a constraint that warrants direct investigation of sperm fate in the female reproductive tract.

Here, we address these questions in the cactophilic *D. arizonae*, the only species for which mdFTP data are currently available [13], and in its sibling, *D. mojavensis*. We identified mdRNA candidates in both species using a heterospecific cross strategy that exploits species-specific transcript sequences as molecular barcodes, allowing highly specific accounting of male-derived transcripts in the lower female reproductive tract (Fig 1B). We then characterized the likely tissue of origin of mdRNA candidates by analyzing their expression in testes, sperm-filled seminal vesicles, and male accessory glands (together with the ejaculatory duct and bulb) using both whole-transcript and 3’-end RNA-seq data. We further examined the functional properties of mdRNA candidates through protein domain enrichment analysis. As sperm appeared as a potentially major vehicle for mdRNAs, we generated a transgenic line with GFP-tagged sperm to track the fate of sperm-associated mRNAs within the female reproductive tract.

## Results and discussion

### Identification of mdRNA candidates

We applied a stringent four-filter pipeline to RNA-seq data from heterospecific crosses between *D. arizonae* and *D. mojavensis* [25,26] to identify mRNAs transferred from males to the female lower reproductive tract (LRT) during mating. The pipeline exploits interspecific sequence divergence as a molecular barcode: *D. arizonae* transcripts detected in mated *D. mojavensis* female reproductive tracts must originate from the *D. arizonae* male, and vice versa. No candidates were identified in the female head tissue from either cross, confirming the pipeline's specificity. The number of candidate genes passing each filter individually, as well as those passing increasing combinations of filters, is shown in Fig 2A and S1–S7 Tables.

**Fig 2:**
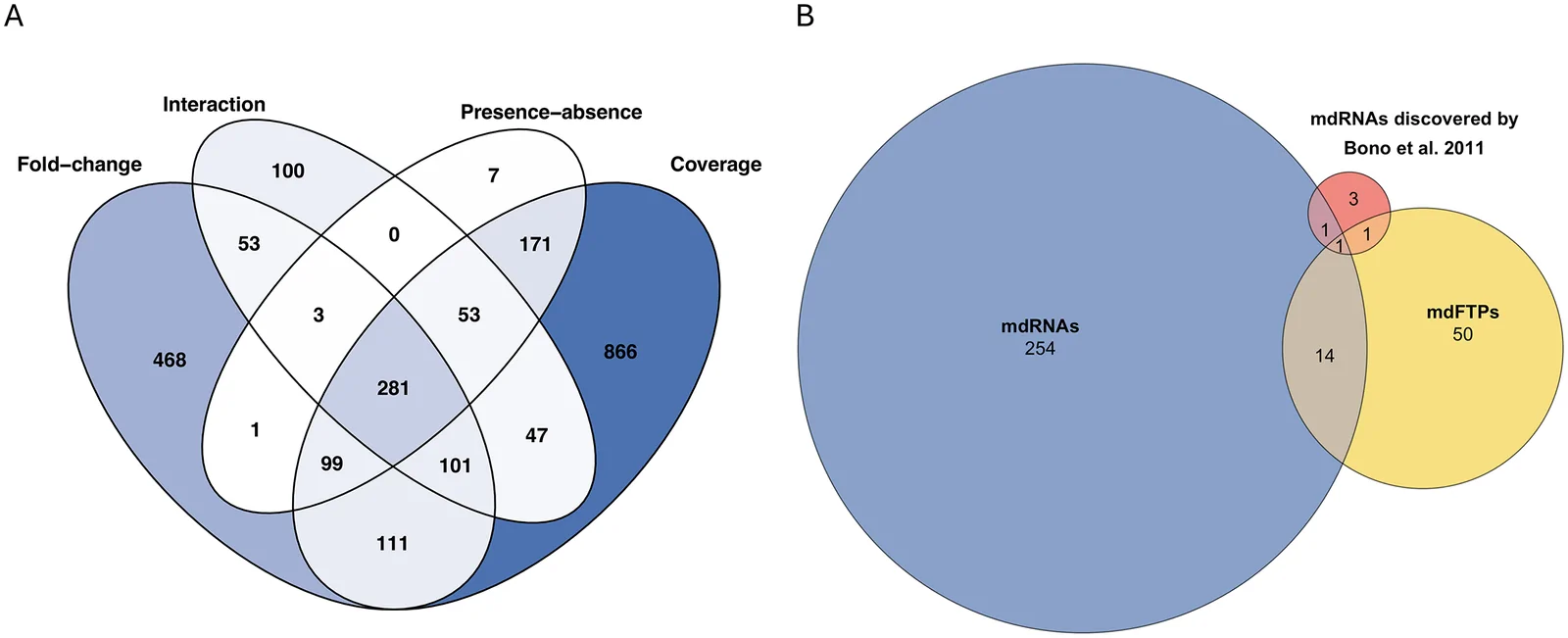
Identification and validation of mdRNA candidates. (A) Venn diagram showing the number of candidate genes passing each of the four mdRNA identification filters for the combined *D. arizonae* and *D. mojavensis* heterospecific crosses: fold-change (significant expression increase of the male isoform in mated females), interaction (significantly higher increase of the male vs. the female isoform in mated females), presence-absence (> 1 TPM of the male isoform and < 1 TPM of the female isoform in mated females), and coverage (> 50% of the male isoform covered by reads in mated females, with coverage increasing at least 4-fold over virgins). For each ortholog pair, the filter results shown correspond to whichever cross direction passed the greater number of filters, with ties resolved in favor of the *D. mojavensis*♀ × *D. arizonae*♂ cross. The intersection of all four filters defines the conservative mdRNA catalog of 281 unique genes before the conspecific exclusion filter. (B) Venn diagrams showing the overlap between the final combined list of 270 mdRNA candidates and two independent validation datasets: 66 genes encoding mdFTPs in *D. arizonae* [13] and 6 mdRNAs confirmed by RT-PCR in *D. mojavensis* [8].

As an additional specificity check, we applied the same four-filter pipeline to conspecific crosses, which identified 11 *D. arizonae* and 0 *D. mojavensis* genes passing all four filters. Because these genes show the mdRNA signature even in the absence of a heterospecific male, they cannot represent true male-to-female transfer detectable by our interspecific barcoding approach, and likely instead reflect cases of insufficient interspecific isoform divergence or highly variable mating-responsive expression. We therefore applied a conspecific exclusion filter, removing any gene that passed all four filters in the conspecific cross from the corresponding heterospecific candidate list, eliminating these 11 *D. arizonae* candidates.

The final catalog comprises 228 *D. arizonae* mdRNA candidates and 83 *D. mojavensis* mdRNA candidates, of which 41 are orthologous pairs that passed filters in both species, yielding 270 unique mdRNA-encoding genes in the combined catalog (S1–S7 Tables). To validate pipeline performance, we cross-referenced the mdRNA catalog with two independent datasets. Of 66 genes currently annotated in the *D. arizonae* genome that encode mdFTPs [13], 39 (59%) passed at least one filter and 15 (23%) passed all four filters in at least one cross direction (Fig 2B). Of the 6 annotated *D. arizonae* genes confirmed as mdRNAs by Bono et al. [8] via RT-PCR, 4 (67%) passed at least one filter and 2 (33%) passed all four filters (Fig 2B).

These recovery rates reflect the deliberately conservative design of our four-filter pipeline (see Materials and Methods), which prioritizes specificity over sensitivity: rather than producing a comprehensive but noisy list, we aimed for a high-confidence catalog, accepting that some true mdRNAs would not be recovered in exchange for minimizing false positives. It is also possible that some mdFTP candidates not recovered here represent false positives in the original proteomic dataset. Given the low sensitivity of our pipeline, recovering only 23% of known mdFTPs and 33% of Bono et al. [8] candidates, the true mdRNA repertoire in these species certainly exceeds the 270 candidates identified here.

The incomplete recovery expected from our conservative pipeline may, at least in part, account for the limited observed overlap between species (41 shared candidates). However, the marked difference in candidate numbers between the two cross directions (228 versus 83) warrants consideration. Several factors may contribute to this asymmetry. First, *D. arizonae* males may transfer more ejaculate to heterospecific *D. mojavensis* females than vice versa, resulting in higher concentrations of male-derived transcripts in the female reproductive tract and consequently greater power to detect them. Second, evolutionary divergence in mdRNA repertoires between the species could also contribute to the observed difference in candidate numbers. Third, segregating polymorphisms at sites that distinguish the two species’ isoforms could affect detection sensitivity asymmetrically between the two cross directions. These factors possibly act in combination, and their relative contributions cannot be resolved with the current data.

### mdRNA candidates show expression consistent with production in multiple male reproductive tract compartments

Tissue-of-origin analyses were restricted to the 228 *D. arizonae* mdRNA candidates, which include 41 shared candidates, as this species yielded substantially more candidates than *D. mojavensis* (228 versus 83), warranting more robust comparisons.

Testes, the male accessory glands, and the ejaculatory bulb and duct are responsible for generating the bulk of the cellular and seminal fluid components transferred to females during mating and represent the most likely sources of mdRNAs (Fig 1A). Therefore, to characterize the likely tissue of origin of the 228 *D. arizonae* mdRNA candidates, we compared their abundance in the heterospecifically mated female LRT with their expression in testes and in the male accessory glands together with the ejaculatory bulb and duct (hereafter MAGs), using an available whole-transcript RNA-seq dataset for both tissues. Spearman's rank correlation between LRT abundance and tissue expression was significant for both organs: testis ρ = 0.463 (p < 0.001) and MAG ρ = 0.386 (p < 0.001) (Fig 3A). These correlations indicate that mdRNA candidates tend to be more abundantly detected in the female LRT when they are more highly expressed in either male organ. However, numerous low-abundance male transcripts were also detected in females, indicating that high expression in the male reproductive tract is not required for transfer.

**Fig 3:**
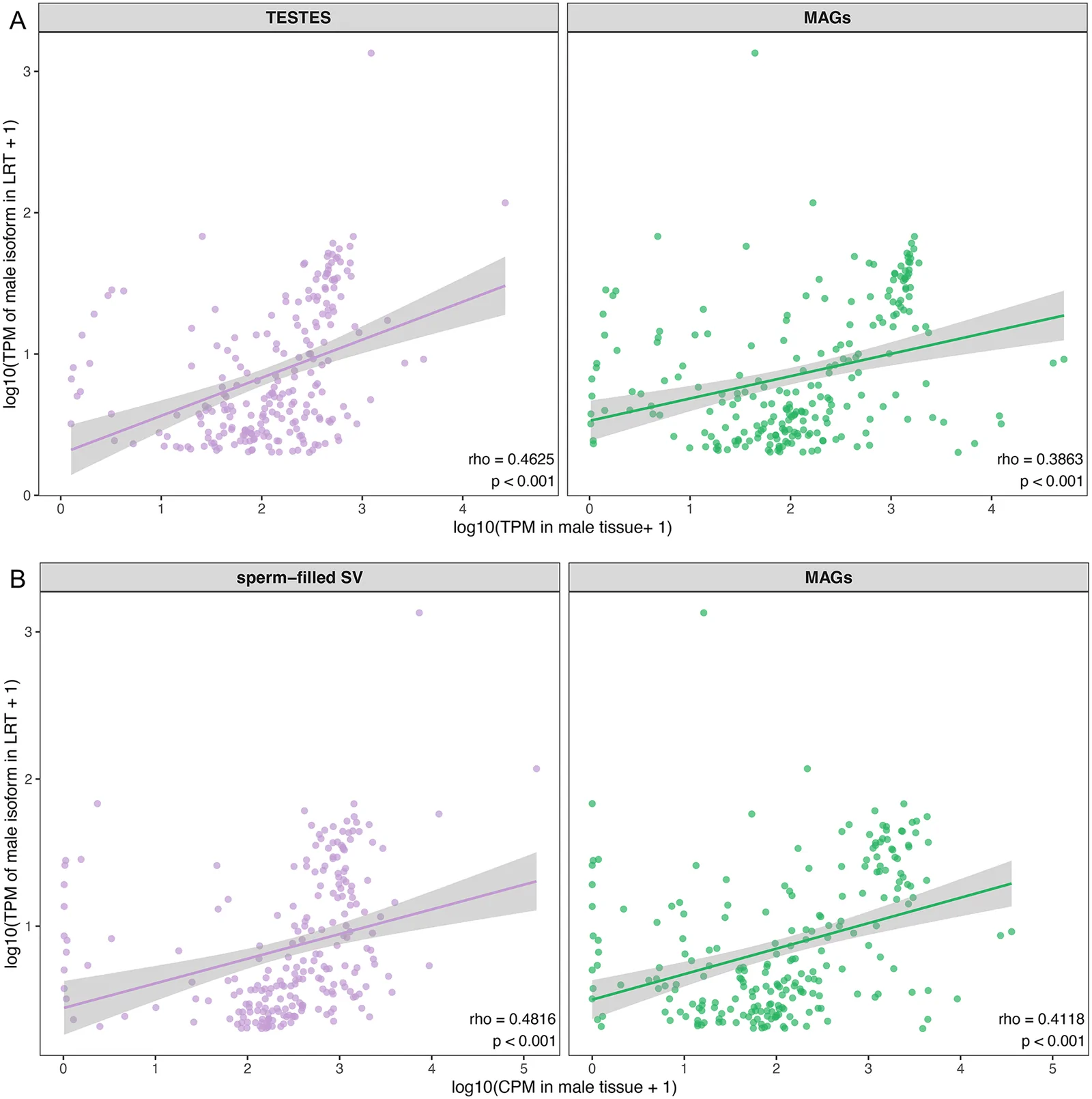
Scatter plots showing the relationship across the 228 *D. arizonae* mdRNA candidates between mdRNAs’ abundance in the LRT of heterospecifically mated females and gene expression in (A) testes (left) and MAGs (right), or in (B) sperm-filled SVs (left) and MAGs (right). Spearman's rank correlation coefficients and p-values are shown for each comparison along with a regression line and its 95% confidence interval (grey envelope).

Because testes contain pre-meiotic germline and somatic cells in addition to mature sperm, and because we wished to compare sperm-specific expression directly against MAGs using a replicated, unique sequencing approach, we generated a new 3’-end RNA-seq dataset from sperm-filled seminal vesicles (SVs) and MAGs. Across all samples (3 for SVs and 3 for MAGs), sequencing depth averaged 19.90 million reads per sample (range: 19.14–20.48). We repeated the Spearman rank correlation analysis using this new dataset to examine whether mdRNA abundance in the mated female LRT also correlates with expression in SVs and MAGs. Correlations were significant for both tissues: SV ρ = 0.482 (p < 0.001) and MAG ρ = 0.412 (p < 0.001) (Fig 3B), closely mirroring the pattern observed in the whole-transcript testis data. Equivalent analyses performed on the combined 270-candidate catalog anchored on *D. arizonae* identifiers yielded qualitatively identical results (S1 Fig).

The similar correlations observed between each male organ and the female LRT may partly reflect shared expression patterns across male reproductive tissues rather than independent tissue-of-origin signals. Indeed, we found strong positive correlations between expression in testes and MAGs (whole-transcript dataset: ρ = 0.76, p < 0.001), and between sperm-filled SVs and MAGs (3′-end dataset: ρ = 0.508, p < 0.001). To more directly assess whether tissue-of-origin predicts mdRNA abundance in the mated female LRT, we examined the relationship between organ-specificity of expression and LRT abundance using both datasets (Fig 4). In the 3′-end dataset, we classified the 228 *D. arizonae* mdRNA candidates into SV-biased (log2(SV/MAG) ≥ 2; n = 90), MAG-biased (log2(SV/MAG) ≤ −2; n = 11), and non-biased (n = 127) groups. In the whole-transcript dataset, the equivalent groups were testis-biased (n = 40), MAG-biased (n = 19), and non-biased (n = 169). In both datasets, tissue-biased candidates from sperm-associated tissues and MAGs showed similar median LRT abundance (3′-end: 3.07 vs 2.65 TPM, p = 0.926; whole-transcript: 4.13 vs 2.65 TPM, p = 0.188), suggesting that neither organ contributes disproportionately to the transferred mdRNA pool. Sperm-associated tissues consistently yielded more tissue-biased candidates than MAGs, which could reflect the more specialized transcriptome of sperm, a genuinely larger number of sperm-specific mdRNAs, or both.

**Fig 4:**
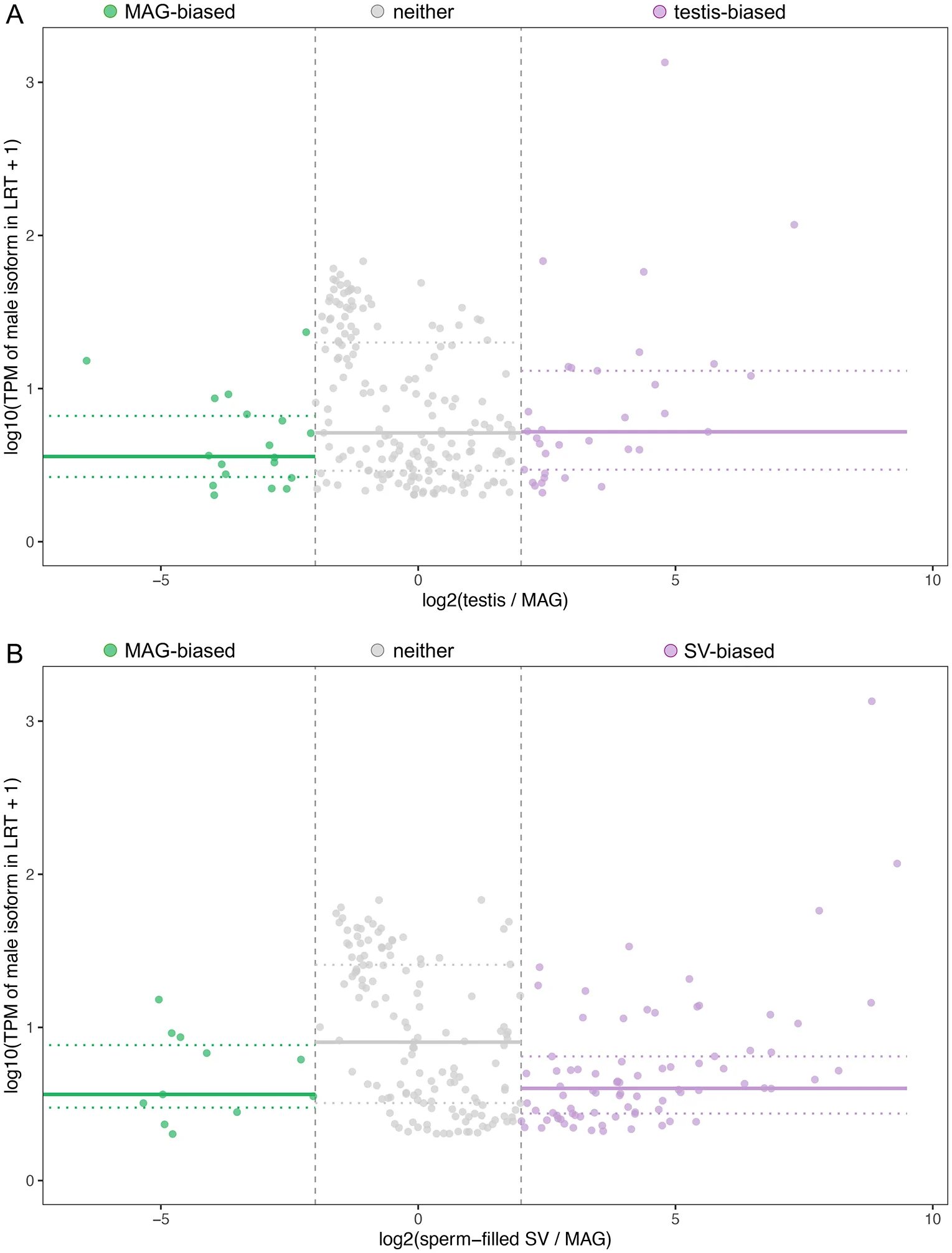
Relationship between mdRNA abundance in the heterospecifically mated female LRT and tissue-of-origin specificity for (A) the whole-transcript RNA-seq dataset, with the x-axis showing testis vs MAG specificity, and (B) the 3′-end RNA-seq dataset, with the x-axis showing sperm-filled SV versus MAG specificity. Each point represents one mdRNA candidate. The x-axis shows the log2 ratio of expression in the sperm-associated tissue versus MAGs, where positive values indicate higher expression in the sperm-associated tissue and negative values indicate higher expression in MAGs. The y-axis shows the log10(TPM + 1)-transformed abundance of the male isoform detected in the LRT. Points are colored by tissue specificity: purple for candidates with ≥ 4-fold higher expression in the sperm-associated tissue, green for candidates with ≥ 4-fold higher expression in MAGs, and grey for candidates with similar expression in both tissues. Dashed vertical lines mark the 4-fold threshold. Horizontal lines show the median (solid) and interquartile range (dotted) of LRT abundance for each group.

To further characterize the tissue of origin, we assessed whether the mdRNA candidates most abundant in the mated female LRT also tend to be most abundant in each male tissue, by computing the overlap between the top 10% most abundant candidates in the LRT and the top 10% most highly expressed candidates in each tissue. Overlap with MAGs was consistent across both whole-transcript (34.8%, 8/23) and 3′-end (34.8%, 8/23) datasets, indicating that a substantial fraction of mdRNAs abundant in the LRT are also highly expressed in the accessory glands. In contrast, overlap was higher with whole testes (39.1%, 9/23) than with sperm-filled SVs (26.1%, 6/23), suggesting that some abundant mdRNAs could originate from spermatogenic cells or other testis cell types rather than from mature sperm stored in the SV. Taken these results together with the broadly similar correlation strengths observed for sperm-associated tissues and MAGs across both datasets, our results do not favor either organ as the predominant source.

As a complementary approach to assessing tissue of origin, we examined the overlap between *D. arizonae* mdRNA candidates and proteome datasets: the *D. arizonae* sperm proteome [27] and a MAG proteome generated in this study for the same species. The sperm proteome comprised proteins encoded by 2,422 genes and the MAG proteome comprised proteins encoded by 1,416 genes (S8 Table). A large and broadly similar fraction of mdRNA candidates was detected in both the sperm proteome (77.2%, 176/228) and the MAG proteome (61%, 139/228), consistent with the transcript-level correlation analyses and further supporting the interpretation that mdRNA candidates have multiple male tissue origins.

### mdRNA candidates encoding mdFTPs show mixed tissue origins

Among the 228 *D. arizonae* mdRNA candidates, 15 encode mdFTPs identified by Matzkin et al. [13], constituting a high-confidence subset with direct functional evidence of translation in the female reproductive tract. We examined the tissue expression profiles of these 15 genes to determine whether mdFTP-encoding genes have a distinctive origin compared to the broader catalog.

Using the 3’-end RNA-seq data from SVs and MAGs, the 15 high-confidence mdFTP genes showed a median log2(SV/MAG) ratio near zero (0.03), reflecting a clearly bimodal distribution (Fig 5). Four genes were highly SV-enriched, with *ARI19798* showing the strongest SV bias (log2 = 5.40, corresponding to 2,114 CPM in SVs vs. 49 CPM in MAGs), while four genes were highly MAG-enriched, with *ARI20988* and *ARI23381* showing extreme MAG dominance (>27,000 and >35,000 CPM in MAGs, respectively). Four other genes exhibited similar expression levels in both tissues, and the remaining three genes had near-zero CPM in both, possibly reflecting expression in cell types underrepresented in bulk sequencing. Using the whole-transcript testis/MAG dataset, the same 15 genes showed a highly similar bimodal pattern — including the same standout testis- and MAG-enriched genes (*ARI19798*, *ARI20988*, and *ARI23381*) — though with an overall MAG-skewed median (log2(testis/MAG) = −2.01).

**Fig 5:**
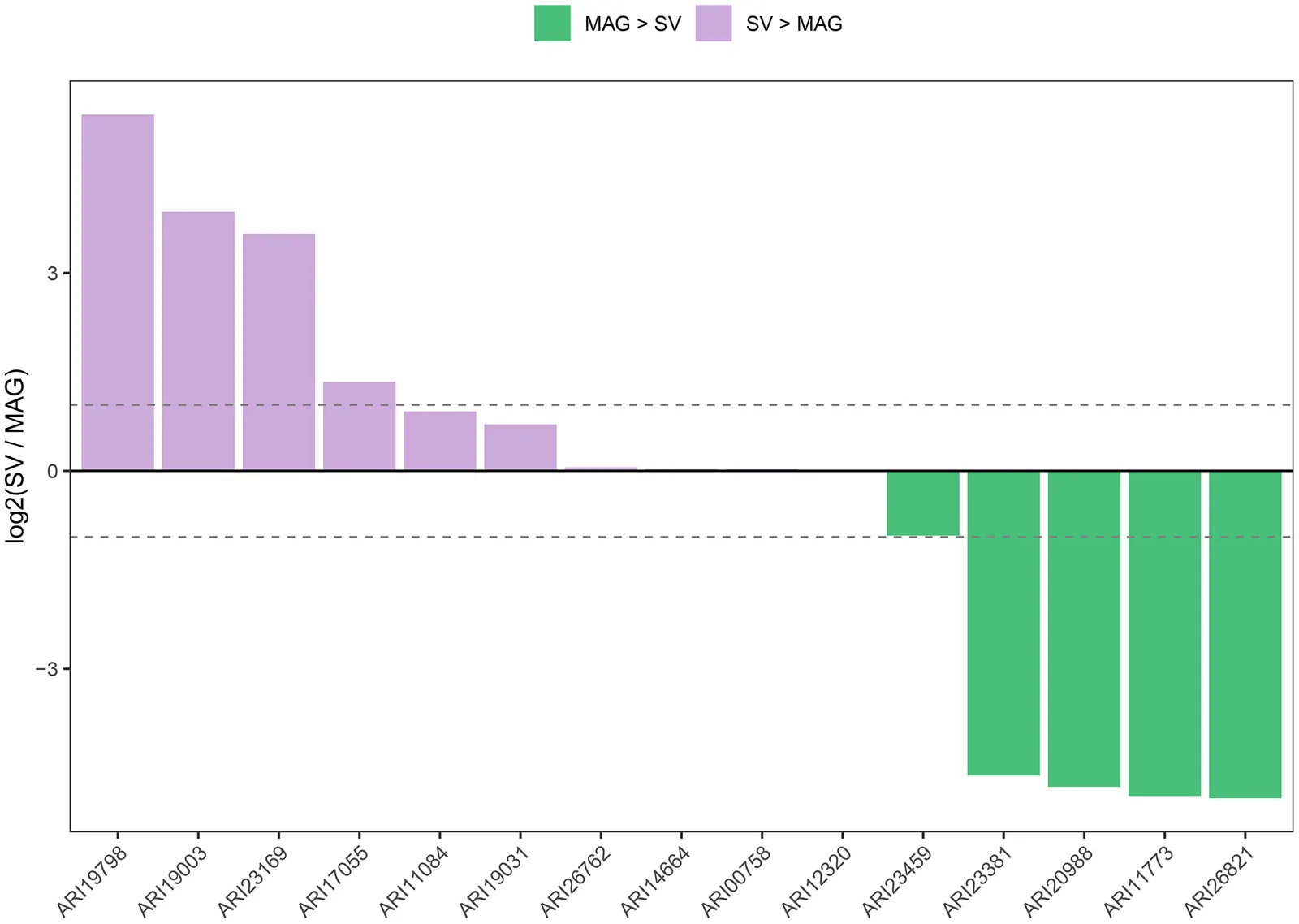
Log2-transformed ratio of expression (CPM) in sperm-filled SVs relative to MAGs for the 15 high-confidence mdFTP-encoding genes. Genes with log2(SV/MAG) > 0 are SV-enriched, and genes with log2(SV/MAG) < 0 are MAG-enriched. The median log2(SV/MAG) ratio across the 15 genes is 0.03.

These results suggest that mdFTP genes have heterogeneous tissue origins: some are more likely sperm-derived, while others, including some of the most highly MAG-expressed genes, likely originate from the MAGs. Given this heterogeneity, different mdFTPs may enter the female reproductive tract via distinct mechanisms, although our data do not allow us to assign a definitive tissue of origin for individual genes, and resolving this will require additional experimental approaches.

### Functional enrichment of mdRNA candidates

Functional enrichment analyses were performed on the combined catalog of 270 mdRNA candidates, anchored on *D. arizonae* gene identifiers, with *D. mojavensis*-specific candidates represented by their *D. arizonae* orthologs. This approach maximizes the number of candidates available for enrichment analysis and is justified by the close evolutionary relationship between the two species, which makes functional annotation largely interchangeable between orthologs.

Over-representation analysis of InterPro protein domain annotations revealed a single enriched term among the 270 mdRNA candidates: zinc-binding ribosomal proteins (IPR011332; 4 of 7 annotated genes, enrichment ratio = 25.2, FDR = 0.014). The four genes supporting this term — ARI10618/RpS27A, ARI22486/RpL36A, ARI15627/RpL37A, and ARI21554/RpL37-1 — are highly conserved and encode structural components of the cytoplasmic ribosome. All four genes are present among the 228 *D. arizonae* mdRNA candidates, confirming that this result is not dependent on the inclusion of *D. mojavensis*-specific candidates in the combined catalog.

The overrepresentation of structural components of the ribosome is intriguing. Beyond their canonical role, ribosomal proteins have well-established extra-ribosomal roles, including in transcription, DNA repair, and apoptosis [28]. Their presence among transferred mdRNAs raises the possibility that male-derived ribosomal protein transcripts could influence gene expression or cellular homeostasis in the female reproductive tract, though none of the four genes encode confirmed mdFTPs, so direct functional evidence is currently lacking.

Notably, the enrichment for ribosomal protein transcripts is consistent with findings from Fischer et al. [14], who reported that mRNAs encoding ribosomal proteins are a conserved and abundant component of the spermatozoal mRNA repertoire in both *Drosophila* and humans. This convergence lends additional support to the hypothesis that a substantial fraction of mdRNA candidates, including those enriched for ribosomal protein transcripts, are sperm-derived. Fischer et al. [14] used YFP-tagged reporter lines for several of the most abundant *Drosophila* spermatozoal transcripts, including the ribosomal protein genes *RpS9* and *RpL41*, to demonstrate directly that paternal sperm mRNAs are transferred to the oocyte at fertilization and remain detectable in the embryo before the onset of zygotic transcription. If the ribosomal-protein-enriched mdRNAs identified here follow a similar route, they could be positioned to influence early gene expression in the fertilized egg, paralleling the broader functional role proposed for sperm-borne RNA in shaping early development [15]. However, given the small number of genes involved and the absence of other enriched functional categories, caution is warranted in interpreting this result.

### Conservation of mdRNA candidates across *Drosophila* species

We used OrthoFinder to identify mdRNA orthologs among *D. arizonae*, *D. mojavensis* (diverged from *D. arizonae* ~1 MYA), *D. virilis* (diverged from *D. arizonae* 20–30 MYA), and *D. melanogaster* (diverged from *D. arizonae* 30–40 MYA). To assess their evolutionary conservation, we examined whether orthogroups containing *D. arizonae* or *D. mojavensis* mdRNA candidates also included genes identified as seminal RNA candidates in *D. melanogaster* [12] or *D. virilis* [11]. The 270 *D. arizonae*/*D. mojavensis* mdRNA candidates were grouped in 263 orthogroups. The vast majority of them, 234 (86.7%), did not include *D. melanogaster* or *D. virilis* mdRNA candidates; 27 (10%) had *D. melanogaster* mdRNAs; 2 (0.7%) had *D. virilis* mdRNAs, and none had candidates of both species (Fig 6; S9 Table). When interpreting these comparisons, it is important to note an asymmetry between the two reference datasets: the *D. melanogaster* mdRNA list of Cridland et al. [12] is likely to be comparatively comprehensive, as candidates were identified across multiple samples and conditions in that species, whereas the *D. virilis* dataset of Ahmed-Braimah et al. [11] is considerably smaller and less exhaustive.

**Fig 6:**
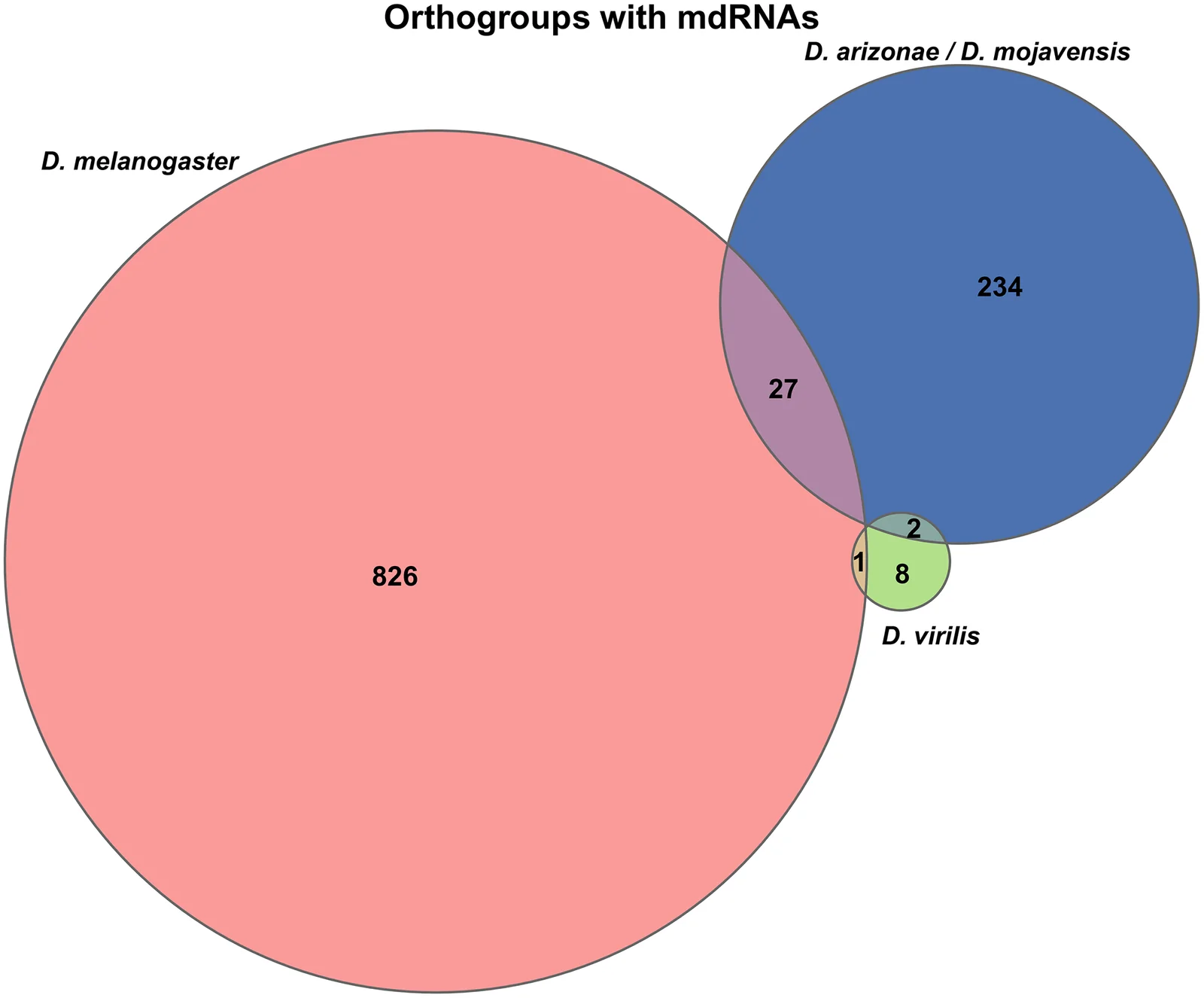
Venn diagram showing conservation of mdRNA candidates across *Drosophila* species. The overlap is at the level of orthogroups, between the 270 mdRNA candidates (grouped into 263 orthogroups) and seminal RNA candidates identified in *D. melanogaster* [12] and the *D. virilis* species complex [11].

To assess whether the overlap with *D. melanogaster* mdRNAs exceeds chance expectation, we performed a hypergeometric test using the 10,907 orthogroups containing genes of all three species as the background, the only orthogroups where overlap is possible. The observed overlap of 27 orthogroups was 1.32-fold higher than the expected 20.6 by chance, but this enrichment was not statistically significant (p = 0.089), suggesting that the two catalogs do not share more orthogroups than expected given their respective sizes. This limited overlap likely reflects a combination of different filtering criteria and barcoding sensitivity between studies, genuine divergence in which transcripts are transferred across the approximately 40 million years separating these species groups, and potential differences in the completeness of each catalog. Despite this, the two studies converge on a similar picture of tissue origin: Cridland et al. [12] found that male-derived transcripts in *D. melanogaster* have heterogeneous tissue origins, with most showing expression consistent with a sperm origin alongside a smaller subset enriched for accessory-gland secondary-cell markers, a pattern that closely parallels our findings. This convergence suggests that even when the specific transcripts transferred have diverged, the broader tissue-of-origin landscape of mdRNA transfer may be conserved across *Drosophila* species.

### Transferred sperm become trapped in the insemination reaction mass, a potential route for RNA release

Given the high likelihood that many mdRNAs are of sperm origin and our previous results showing translation of male RNA into mdFTPs, we consider potential mechanisms by which sperm RNA could be released from within sperm. To investigate the fate of transferred sperm within the female reproductive tract, we generated a *D. arizonae* line expressing a protamine-GFP fusion in sperm nuclei and imaged the lower reproductive tract of 6–12 mated females live at each of three time points: within 15 min (0 h), 4 h, and 8 h postmating (Fig 7). At all time points, GFP-labeled sperm were predominantly detected in the seminal receptacle, the female's sole sperm storage organ in this species (Fig 1B). However, at the first two time points (0 and 4 h), hundreds of sperm cells were also observed within the insemination reaction mass, a transient uterine structure formed from coagulated ejaculate components during copulation that is characteristic of some species in the *repleta* group and absent in the *melanogaster* group [29]. Sperm within the seminal receptacle were motile, consistent with active, functional storage, while those trapped within the insemination reaction mass showed no movement and no apparent change in their condition over the first 4 h. At these time points, sperm heads within the insemination reaction mass were too densely aggregated to resolve individually; however, by using the size of isolated sperm heads visible at the periphery of the mass as a reference, the overall size of the fluorescent aggregate was consistent with the presence of 200–500 sperm cells in all cases examined.

**Fig 7:**
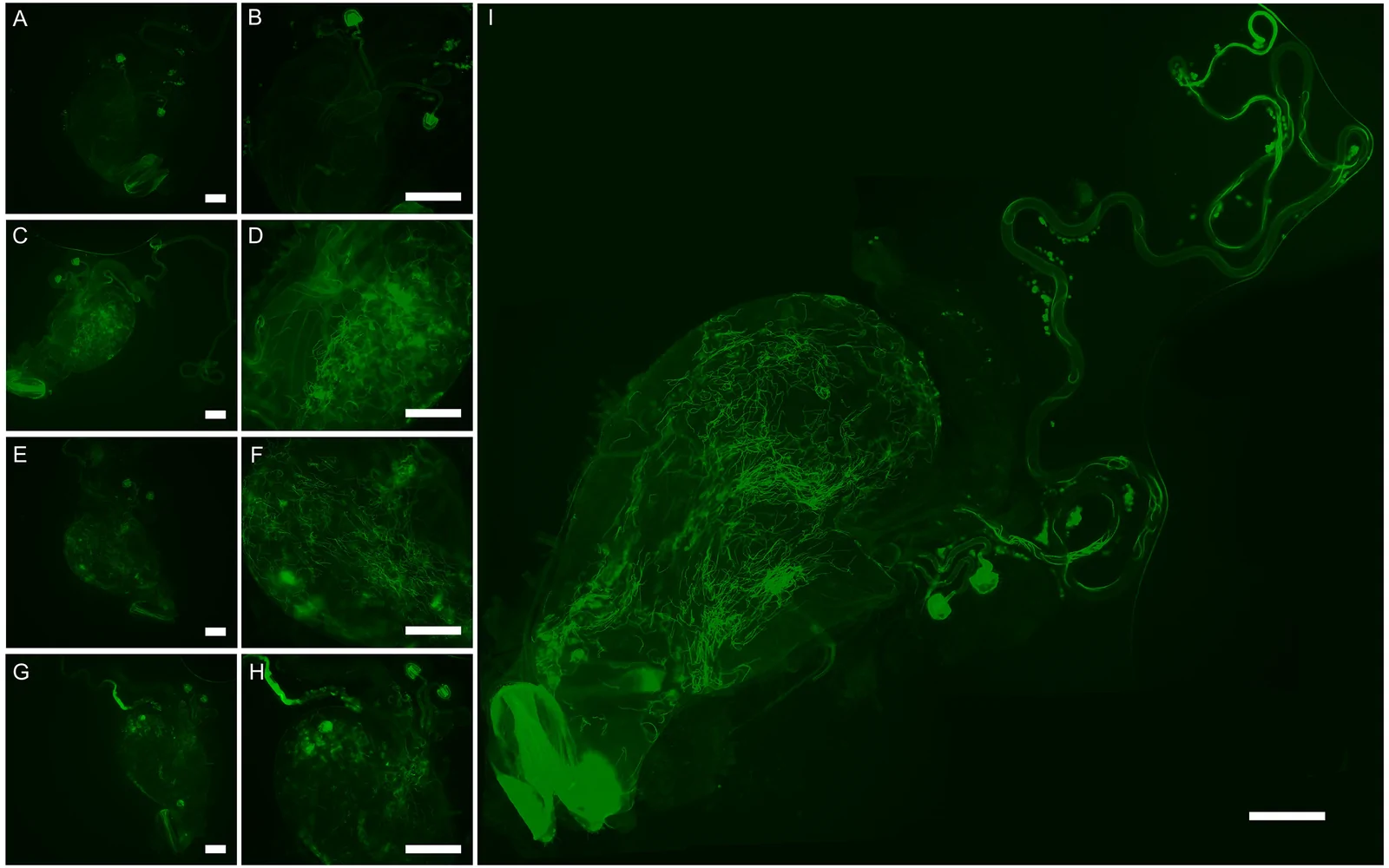
Fate of GFP-labeled sperm in the female lower reproductive tract following mating. Representative live fluorescence microscopy images of the LRT of *D. arizonae* females. (A–B) Virgin female imaged as a negative control; no GFP signal is detected beyond autofluorescence of some external fat body cells and the sclerotized ovipositor and spermatheca. (C–H) Mated females dissected at 0 h (C–D), 4 h (E–F), and 8 h (G–H) after the end of copulation. Within each time point, the left image shows the whole LRT at 100 × magnification (except for the apical portion of the seminal receptacle, which could not always be captured within the field of view), and the right image shows a closer view of the uterus at 200 × magnification, with non-motile GFP-labeled sperm visible within the insemination reaction mass. (I) Composite image of the whole LRT at 15 min after the end of copulation, assembled with Photoshop from multiple frames at 400 × magnification, showing GFP-labeled sperm at both locations: the uterus and the seminal receptacle. Scale bar = 100 µm.

By 8 h postmating, hundreds of motile sperm were still observed in the seminal receptacle of all females. In half of the dissected tracts (6/12), the insemination reaction mass was no longer evident, suggesting that it had fully or partially dissolved or been expelled within this window. In these six cases, only a small number of non-motile sperm remained in the uterus, and given the reduced density, individual cells could be resolved and counted, yielding counts of 4, 6, 17, 25, 29, and 30. In the remaining 6 females, aggregates of more than 100 sperm persisted within the reaction mass.

To our knowledge, this is the first direct observation of non-motile sperm persisting within the insemination reaction mass. During this window, sperm cells could undergo lysis, potentially releasing their RNA contents into the female reproductive tract lumen. Given that a substantial fraction of our mdRNA candidates, particularly those encoding mdFTPs, show expression compatible with a sperm origin, the persistence of immotile, trapped sperm that may be dying in the uterus for more than 4 h after mating suggests a possible route by which these mdRNAs could exit sperm cells and become accessible to female cells. It should be noted, however, that our expression data cannot distinguish mdRNAs carried within sperm cells from those transferred in the seminal fluid or those associated with the sperm surface (possibly within vesicles), which would not require sperm lysis. Consequently, we cannot confirm that any mdFTP-encoding mdRNA is actually carried within sperm, and the entire argument above is contingent on that assumption. The observation reported here is therefore best interpreted as a novel and plausible hypothesis rather than an established mechanism, and testing it directly will require further experiments such as sperm RNA sequencing and direct localization of mdRNA transcripts to the insemination reaction mass at different time points.

## Materials and methods

### mdRNA identification

Throughout this manuscript, we refer to *D. arizonae* and *D. mojavensis* by species name rather than by isofemale line designation (ARI and MOV, respectively), for ease of reading. Data processing and analysis were performed using R v4.4.1 [30] and Python v3.7.12.

RNA-seq data were obtained from Diaz et al. [25,26], who profiled the head and lower reproductive tract (LRT), respectively, of *D. mojavensis* females mated to *D. arizonae* males (MOV♀ × ARI♂) and *D. arizonae* females mated to *D. mojavensis* males (ARI♀ × MOV♂), along with conspecifically mated and virgin females of each species as controls. LRTs were dissected from mated females at 45 minutes postmating, alongside virgin controls. Complete descriptions of RNA extraction, library preparation, and sequencing procedures can be found in Diaz et al. [25,26]. This work comprised 11–34 million paired-end short reads (150 bp) per sample. Raw reads were trimmed using fastp v0.23.4 [31] with default parameters (except cut_tail_mean_quality = 25 and length_required = 75) and aligned to a combined reference containing both species’ transcriptomes (only the longest transcript was retained per gene) using STAR v2.7.11 [32] with near-perfect match alignment parameters (outFilterMismatchNmax = 0, outFilterMismatchNoverReadLmax = 0, outFilterMultimapNmax = 1, outFilterScoreMinOverLread = 0.99, outFilterMatchNminOverLread = 0.99, alignEndsType = Local), designed to hinder cross-species alignments. The reference was generated from the genome assemblies and annotations reported by Benowitz et al. [33]. Transcript-level quantification was performed with Salmon v1.10.0 [34]. Because the two species have diverged sufficiently that each species’ transcripts map preferentially to their own genome, species-specific isoforms serve as molecular barcodes: *D. arizonae* transcripts detected in *D. mojavensis* females mated to *D. arizonae* males must originate from the *D. arizonae* male, and vice versa.

Differential expression analysis was performed using edgeR [35] with a generalized linear model including mating status (mated vs. virgin), isoform species (*D. arizonae* vs. *D. mojavensis*), and their interaction term. The interaction term captures male-isoform transcripts that increase disproportionately in the mated female relative to the increase in the virgin female, the expected signature of male-transferred RNA. For each ortholog pair, we tested the male isoform fold-change increase in the mated female and the interaction between mating status and isoform identity. Only one-to-one orthologs identified by OrthoFinder v2.5.5 [36] across *D. arizonae* and *D. mojavensis* proteomes were included in the analysis, and only one protein (the one encoded by the longest transcript) was used to feed OrthoFinder.

Candidate mdRNAs were identified by applying four sequential filters to each ortholog pair. First, the fold-change filter required a significant increase in the male isoform from virgin to mated females (log2FC > 0, FDR < 0.05). Second, the interaction filter required the interaction term to be significant (FDR < 0.05) and in the correct direction: higher increase of the male isoform relative to the female isoform in mated females (interaction log2FC < 0 for *D. arizonae* donors, > 0 for *D. mojavensis* donors), with an exception when the female isoform was absent (TPM = 0 in both conditions), in which case no p-value could be estimated and only the direction requirement was applied. Third, the presence-absence filter required the male isoform to be at less than 1 TPM, a commonly used detection threshold, in virgin females and detectable (TPM > 1) in mated females. Fourth, the coverage filter required at least 50% of the male-isoform transcript to be covered by reads in mated females, with coverage increasing at least 4-fold over virgins. These filtering criteria were deliberately calibrated to be sufficiently stringent to yield no candidates in the *D. arizonae* or *D. mojavensis* female head tissue from the same crosses, which served as a negative control. Although mdRNAs could theoretically circulate from the LRT to other tissues, detection in the head would require sufficient accumulation to pass all four filters, an *a priori* unlikely scenario that justifies using head tissue as a specificity control.

As an additional control, conspecific crosses were processed identically; any gene passing all filters in the conspecific control was removed from the corresponding heterospecific candidate list (conspecific exclusion filter). These excluded genes likely represent cases where isoform divergence between the two species is insufficient for unambiguous assignment, or genes whose expression in the LRT responds to mating in a highly variable manner that mimics the mdRNA signature.

To validate pipeline performance, we cross-referenced the candidate lists against two independent datasets of putative male-derived transcripts. The first comprises 67 genes encoding mdFTPs identified by Matzkin et al. [13] in *D. arizonae*, of which 66 are currently annotated in the *D. arizonae* genome. The second comprises 7 mdRNAs confirmed via RT-PCR in *D. arizonae* by Bono et al. [8], of which 6 are currently annotated in the *D. arizonae* genome. For each dataset, we determined the proportion of genes passing at least one of the four filters and the proportion passing all four filters.

### Expression of mdRNA candidates in the male reproductive tract

To characterize the likely tissue of origin of mdRNA candidates, we focused on the 228 *D. arizonae* mdRNA candidates and *D. arizonae* tissue expression data, as this species yielded substantially more candidates than *D. mojavensis* (228 versus 83), warranting more robust comparisons. The 228 *D. arizonae* candidates include the 41 orthogroups shared with *D. mojavensis*, so this analysis captures the large majority of the combined catalog. Expression data were obtained from two complementary RNA-seq approaches that differ both in the tissues profiled and in their sequencing strategy. The first is a whole-transcript RNA-seq dataset covering testes and MAGs, generated by Benowitz et al. [33] and available from CactusFlyBase (cactusflybase.arizona.edu; NCBI BioProject ID PRJNA1492703), in which stranded paired-end 150 bp reads are distributed across the full gene body. The second is a newly generated 3’-end RNA-seq dataset covering sperm-filled SVs and MAGs, produced using Plasmidsaurus sequencing services (Illumina NovaSeq X Plus), in which single-end stranded 90 bp reads are restricted to the 3’ poly(A) region, eliminating gene-length bias. Seminal vesicles were used as a proxy for sperm in the 3’-end dataset because attempts to collect pure sperm directly proved technically challenging without causing cell damage that could alter RNA content. As a result, testis expression was assessed only with whole-transcript RNA-seq, while SV expression was assessed only with 3’-end RNA-seq; MAGs were profiled with both approaches. These two datasets were analyzed independently and not directly compared, as their different tissue coverage and capture properties preclude direct expression comparisons.

For whole-transcript RNA-seq, 20 MAG pairs and 20 testis pairs were dissected per sample from 7–10 day-old *D. arizonae* males that were reared and aged with females of similar age in groups of approximately 30 flies in banana-molasses media [37]. A single biological replicate was collected per organ type. Complete descriptions of RNA extraction, library preparation, and sequencing procedures can be found in Benowitz et al. [33]. Transcript-level quantification was performed using Salmon v1.10.0 in selective alignment mode against an index built from the reference transcriptome restricted to the longest transcript per gene.

For the 3’-end RNA-seq, MAG and SV samples were also dissected from 7–10 day-old *D. arizonae* males maintained in groups of approximately 30 flies with females of similar age. Males used for seminal vesicle dissections were kept without females for the four days preceding dissection to ensure that vesicles were fully sperm-filled. Three biological replicates were collected per tissue type, and 15 or 30 flies were dissected per MAG or SV sample, respectively. Total RNA was extracted using the Zymo Research Direct-zol RNA MicroPrep kit following the manufacturer's protocol. Library preparation and sequencing were performed by Plasmidsaurus using their 3’-end mRNA-seq service. Reads were aligned to the *D. arizonae* genome with STAR using a 3’-extended gene annotation (ARI_r1.0-UTRextended.gff) that improves capture of 3’-biased reads. This annotation was generated with a custom script that extends the last exon until the coverage drops by more than 10 times the average coverage of the existing last exon or until it reaches the next annotated gene (CoverageUTRExtend.py). For that, we used bulk RNA-seq libraries available in CactusFlyBase for the ARI line (NCBI BioProject ID PRJNA1492703) to calculate per-strand coverage with bamCoverage from the deepTools package [38]. AGAT v1.0 [39] was then used to repair the ARI_r1.0.gff annotation. Read counts were obtained with featureCounts v2.0.3 [40] using strand-specific counting (-s 1) and multi-mapping reads (-M), and normalized to counts per million (CPM) within each replicate. CPM values were averaged across replicates. CPM rather than TPM was used because the 3’ bias of this data renders gene-length normalization inappropriate; reads accumulate only in the terminal ~400 bp regardless of gene length, such that dividing by full gene length would artificially penalize longer transcripts.

The following analyses were performed for each dataset. First, Spearman's rank correlation was calculated between mdRNA candidate abundance in the heterospecifically mated female LRT and expression in each male tissue. Second, we assessed whether expression in testes/SVs and MAGs is correlated across mdRNA candidates (Spearman's rank correlation), and whether tissue-of-origin specificity predicts LRT abundance: candidates were classified as sperm-associated tissue-biased, MAG-biased, or non-biased using a 4-fold threshold, and LRT abundance was compared across groups using Wilcoxon rank-sum tests. To determine whether differences in LRT abundance between groups were explained by overall male tissue expression levels, total expression (sum of expression in both tissues) was also compared across groups using Wilcoxon rank-sum tests. Third, we computed the overlap between the top 10% most abundant mdRNA candidates in the mated LRT and the top 10% most expressed candidates in each male tissue.

As a complementary approach to characterize the tissue of origin of *D. arizonae* mdRNA candidates, we examined their overlap with sperm and MAG proteomes. The *D. arizonae* sperm proteome was obtained from Bono et al. [27]. Briefly, sperm were collected from 20 virgin sexually mature males by tearing open the seminal vesicles and removing sperm clumps, rinsed twice with Ringer's solution, and subjected to liquid chromatography tandem mass spectrometry at the Central Analytical Mass Spectrometry Facility and W.M. Keck Foundation Proteomics Resource at the University of Colorado Boulder. Proteins were identified and quantified using MaxQuant v2.0.3.0 [41], requiring at least two peptides per protein for identification. Complete descriptions of sperm proteomics procedures can be found in Bono et al. [27]. The MAG proteome was generated from MAGs dissected from virgin sexually mature males of both species following the same proteomics procedures. We then determined the fraction of mdRNA candidates whose encoded proteins were detected in each proteome and assessed enrichment relative to the background (all ortholog pairs) using a one-tailed Fisher's exact test.

To investigate whether mdRNAs encoding mdFTPs have a distinctive tissue expression profile compared to other mdRNA candidates, we identified genes that both passed all four mdRNA identification filters and encode an mdFTP as reported by Matzkin et al. [13]. For these genes, we compared expression levels in testes and MAGs using whole-transcript RNA-seq values, and in sperm-filled seminal vesicles and MAGs using 3’-end RNA-seq CPM values, calculating the log2-transformed testes-to-MAGs ratio and the log2-transformed seminal vesicle-to-MAGs ratio, respectively.

### Functional enrichment analysis

To identify protein domains and functional categories overrepresented among mdRNA candidates, we performed over-representation analysis (ORA) using the WebGestaltR package v0.4.6 [42] with a custom gene set database derived from InterProScan annotations.

Protein domain annotations for *D. arizonae* were generated by running InterProScan v5.66-98.0 on the *D. arizonae* proteome, querying the CDD, PANTHER, Pfam, PRINTS, ProSitePatterns, ProSiteProfiles, SFLD, SMART, SUPERFAMILY, Gene3D, HAMAP, NCBIfam, PIRSF, FunFam, Coils, and MobiDBLite databases. Entries with valid InterPro accession numbers were retained and used to construct a GMT-formatted gene set file, in which each InterPro term defines a set of *D. arizonae* genes annotated with that domain. The background gene set consisted of all *D. arizonae* ortholog pairs (12,910 genes) with at least one InterPro annotation (11,200 genes). Of the 270 mdRNA candidates, 254 had InterPro annotations and were included in the enrichment analysis. ORA was performed using the hypergeometric test with Benjamini-Hochberg FDR correction, retaining terms with FDR < 0.05 and a minimum of 5 annotated genes. To reduce redundancy, enriched terms were clustered based on gene set overlap: terms sharing more than 50% of their genes (Jaccard similarity > 0.5) were grouped into clusters, each represented by the term with the lowest p-value.

### Cross-species conservation of mdRNA candidates

To assess the evolutionary conservation of mdRNA candidates, we examined whether genes in the same orthogroups as *D. arizonae* and *D. mojavensis* mdRNA candidates have been independently identified as seminal-transferred RNAs in other *Drosophila* species. We focused on two published datasets: mdRNAs in *D. melanogaster* [12] and mdRNAs in the *D. virilis* species complex [11]. Both datasets are broadly comparable to our own in that they rely on a similar barcoding logic, distinguishing male- from female-derived transcripts in the mated female reproductive tract based on sequence divergence between distinct genetic backgrounds. Cridland et al. [12] used single-nucleotide polymorphism differences between divergent, highly inbred *D. melanogaster* lines to assign transcripts recovered from the female reproductive tract to a male or female origin. Ahmed-Braimah et al. [11] used heterospecific crosses between *D. novamexicana* and *D. americana*, members of the *D. virilis* species complex, to examine postmating transcriptional changes in the female reproductive tract.

Unlike our pipeline and that of Cridland et al. [12], however, the Ahmed-Braimah et al. [11] study was not designed as a systematic survey of male-to-female RNA transfer. Their candidate seminal RNAs were instead defined as transcripts showing higher expression at 3 h postmating relative to 6 and 12 h, a pattern consistent with progressive dilution or degradation of transferred male RNA, but identified using criteria narrower than a dedicated transfer-detection pipeline. Their candidate list is therefore unlikely to represent a comprehensive catalog of seminal-transferred RNAs in this species complex, and its comparatively small size relative to ours and to Cridland et al. [12] should be interpreted in this light. The three studies also differ in sequencing depth, which may further affect the sensitivity of each candidate list and limit direct comparisons of orthogroup overlap as a measure of conservation. Because Ahmed-Braimah et al. [11] reported gene identifiers based on an older *D. virilis* annotation, we first updated these to the current NCBI RefSeq *D. virilis* annotation (Dvir_AGI_RSII-ME) to enable orthology comparison with our catalog.

Ortholog relationships among *D. arizonae*, *D. mojavensis*, *D. melanogaster*, and *D. virilis* were inferred using OrthoFinder v2.5.5 applied to the predicted proteomes of all four species. Only one protein (the one encoded by the longest transcript) was used to feed OrthoFinder. For each orthogroup containing at least one *D. arizonae* or *D. mojavensis* mdRNA candidate, we recorded whether it also contained a *D. melanogaster* or *D. virilis* candidate from the respective published dataset.

### Sperm fate in the lower reproductive tract

To generate a *D. arizonae* line with GFP-labeled sperm, we inserted an attP landing site into the fifth exon of the *white* gene (ARI10968) using CRISPR/Cas9, generating a white-eyed line, and subsequently used ϕC31 integrase-mediated insertion to introduce a protamine-GFP fusion construct (*pARI-ProtA-EGFP/ARI-minwht-attB*) into the landing site. The resulting line (*attP*{*ARI-ProtA-eGFP/ARI-minwht*} *w*^CRISPR^) expresses a Protamine–EGFP fusion protein in sperm nuclei, confirmed by fluorescence microscopy. Full details of molecular procedures, plasmid construction, and line establishment are provided in S1 File.

To examine the fate of transferred sperm within the female lower reproductive tract, 7–10 days-old virgin *D. arizonae* females were mated to same-age Protamine-GFP sperm males, and the lower reproductive tract was dissected and imaged live at three time points: within 15 minutes after the end of copulation, 4 h postmating, and 8 h postmating. Dissections were performed in Grace insect medium and imaged using an Olympus BX53 microscope equipped with a GFP filter set at 100× or 200 × magnification. For each time point, 6–12 mated females were dissected and imaged. At all timepoints, we observed the presence of GFP-labeled sperm in the uterus, within the insemination reaction mass (a transient structure that forms in the uterus from coagulated seminal fluid and ejaculate components shortly after copulation), and in the seminal receptacle, a coiled tubular organ that serves as the sperm storage site in the female reproductive tract. The number of sperm could not be determined for aggregates of >100 sperm because cell proximity made it challenging to distinguish one sperm cell from another.

## Supporting information

S1 Fig Scatter plots showing the relationship across the 270 mdRNA candidates between mdRNAs’ abundance in the LRT of heterospecifically mated *D. arizonae* females and gene expression in (A) testes (left) and MAGs (right), or in (B) sperm-filled SVs (left) and MAGs (right) of *D. arizonae* males. Spearman's rank correlation coefficients and p-values are shown for each comparison along with a regression line and its 95% confidence interval (grey envelope).
(PDF)

S1 Table *D. arizonae* mdRNA candidates — MOV♀ × ARI♂ LRT. Differential expression analysis of *D. arizonae* (male donor) transcripts in the LRT of *D. mojavensis* females mated to *D. arizonae* males versus virgin *D. mojavensis* females. Genes passing all four filters are mdRNA candidates before the conspecific exclusion filter (n = 239). Column descriptions are as follows and apply to S1–S6 Tables. orthogroup: OrthoFinder orthogroup identifier; ARI_gID: *D. arizonae* gene identifier (CFgn format); ARI_gene_name: *D. arizonae* gene name; ARI_tID: *D. arizonae* transcript identifier used for quantification (longest transcript per gene); MOV_gID: *D. mojavensis* gene identifier (CFgn format); MOV_gene_name: *D. mojavensis* gene name; MOV_tID: *D. mojavensis* transcript identifier used for quantification (longest transcript per gene); male_isoform_TPM_in_mated_females: TPM of the male-donor isoform in mated females; male_isoform_TPM_in_virgin_females: TPM of the male-donor isoform in virgin females; female_isoform_TPM_in_mated_females: TPM of the female isoform in mated females; female_isoform_TPM_in_virgin_females: TPM of the female isoform in virgin females; male_isoform_log2FC_mated_vs_virgin_estimate: log2 fold-change of the male isoform between mated and virgin females; male_isoform_log2FC_mated_vs_virgin_padj: Benjamini-Hochberg adjusted p-value for the fold-change test; species_by_status_interaction_estimate: log2 fold-change for the species × mating-status interaction term; species_by_status_interaction_padj: Benjamini-Hochberg adjusted p-value for the interaction term; male_isoform_coverage_pct_in_mated_females: percentage of the male-isoform transcript length covered by at least one read in mated females; male_isoform_coverage_pct_in_virgin_females: percentage of the male-isoform transcript length covered by at least one read in virgin females; fold_change_filter: TRUE if the male isoform shows a significant increase in mated females (log2FC > 0, FDR < 0.05); interaction_filter: TRUE if the species × mating-status interaction term is significant (FDR < 0.05) and in the correct direction, or if the female isoform is absent (TPM = 0) in both mated and virgin conditions; presence_absence_filter: TRUE if the male isoform is undetectable in virgins (TPM ≤ 1) and detectable in mated females (TPM > 1); coverage_filter: TRUE if ≥ 50% of the male-isoform transcript is covered in mated females and coverage increases ≥ 4-fold over virgins.
(TXT)

S2 Table *D. arizonae* mdRNA candidates: negative control — MOV♀ × ARI♂ head. Same analysis as S1 Table applied to head tissue from the same crosses. Head tissue serves as a negative control; no genes are expected to pass all four filters (n = 0). For column descriptions, see S1 Table.
(TXT)

S3 Table *D. arizonae* mdRNA candidates: conspecific exclusion control — MOV♀ × MOV♂ LRT. Analysis of *D. arizonae* isoform signatures in the LRT of *D. mojavensis* females mated to *D. mojavensis* males (conspecific cross). Because no *D. arizonae* male is present, any gene passing all four filters reflects a false positive rather than true male-to-female RNA transfer. Genes passing all four filters are excluded from the final mdRNA catalog (n = 11 excluded). For column descriptions, see S1 Table.
(TXT)

S4 Table *D. mojavensis* mdRNA candidates — ARI♀ × MOV♂ LRT. Differential expression analysis of *D. mojavensis* (male donor) transcripts in the LRT of *D. arizonae* females mated to *D. arizonae* males versus virgin *D. arizonae* females. Genes passing all four filters are *D. mojavensis* mdRNA candidates before the conspecific exclusion filter (n = 83). For column descriptions, see S1 Table.
(TXT)

S5 Table *D. mojavensis* mdRNA candidates: negative control — ARI♀ × MOV♂ head. Same analysis as S4 Table applied to head tissue from the same crosses. Head tissue serves as a negative control; no genes are expected to pass all four filters (n = 0). For column descriptions, see S1 Table.
(TXT)

S6 Table *D. mojavensis* mdRNA candidates: conspecific exclusion control — ARI♀ × ARI♂ LRT. Analysis of *D. mojavensis* isoform signatures in the LRT of *D. arizonae* females mated to *D. arizonae* males (conspecific cross). Because no *D. mojavensis* male is present, any gene passing all four filters reflects a false positive rather than true male-to-female RNA transfer. Genes passing all four filters are excluded from the final mdRNA catalog (n = 0 excluded). For column descriptions, see S1 Table.
(TXT)

S7 Table Combined mdRNA catalog. All one-to-one *D. arizonae*–*D. mojavensis* ortholog pairs, with filter flags from both heterospecific crosses (ARI-donor and MOV-donor) and the final post-exclusion candidate status. All filter flags except the last column (post_exc_mdRNA_candidate) are pre-conspecific-exclusion. For descriptions of ID columns, see S1 Table. Other column descriptions are as follows. ARI_mdFTP: TRUE if the ARI gene encodes a male-derived female-translated protein (mdFTP); mojcluster_MAG: TRUE if the gene is expressed in the male accessory gland (MAG) proteome of the *D. mojavensis* species cluster; mojcluster_SPERM: TRUE if the gene is expressed in the sperm proteome of the *D. mojavensis* species cluster; ARI_fold_change_filter: fold-change filter result for the ARI-donor cross; ARI_interaction_filter: interaction filter result for the ARI-donor cross; ARI_presence_absence_filter: presence-absence filter result for the ARI-donor cross; ARI_coverage_filter: coverage filter result for the ARI-donor cross; ARI_mdRNA_pre_exclusion: TRUE if the gene passed all four filters in the ARI-donor cross (pre-conspecific exclusion); MOV_fold_change_filter: fold-change filter result for the MOV-donor cross; MOV_interaction_filter: interaction filter result for the MOV-donor cross; MOV_presence_absence_filter: presence-absence filter result for the MOV-donor cross; MOV_coverage_filter: coverage filter result for the MOV-donor cross; MOV_mdRNA_pre_exclusion: TRUE if the gene passed all four filters in the MOV-donor cross (pre-conspecific exclusion); post_exc_mdRNA_candidate: TRUE if the gene is in the final combined mdRNA catalog after conspecific exclusion.
(TXT)

S8 Table Sperm and male accessory gland proteomes of *D. arizonae* and *D. mojavensis.* Proteins identified by LC-MS/MS in sperm and MAG proteomes of *D. arizonae* and *D. mojavensis*. The sperm proteome was obtained from Bono et al. [27], and the MAG proteome was generated in this study. Each row corresponds to one one-to-one *D. arizonae*–*D. mojavensis* ortholog pair. For descriptions of ID columns, see S1 Table.
(TXT)

S9 Table Cross-species overlap analysis for mdRNA candidates. Orthogroups containing at least one mdRNA candidate from any of the four species included in the OrthoFinder analysis (*D. arizonae*, *D. mojavensis*, *D. melanogaster*, and *D. virilis*). Each row corresponds to one orthogroup with at least one mdRNA candidate. Column descriptions are provided as follows. category: overlap category indicating which species’ mdRNA candidate lists are represented in this orthogroup; orthogroup: OrthoFinder orthogroup identifier; Dmel_all_genes: all *D. melanogaster* genes in this orthogroup (semicolon-separated FlyBase identifiers); Dvir_all_genes: all *D. virilis* genes in this orthogroup (semicolon-separated NCBI identifiers); ARI_all_genes: all *D. arizonae* genes in this orthogroup (semicolon-separated CFgn identifiers); MOV_all_genes: all *D. mojavensis* genes in this orthogroup (semicolon-separated CFgn identifiers); Dmel_mdRNAs: *D. melanogaster* genes in this orthogroup identified as mdRNA candidates by Cridland et al. [12]; Dvir_mdRNAs: *D. virilis* genes in this orthogroup identified as mdRNA candidates by Ahmed-Braimah et al. [11]; ARI_mdRNAs: *D. arizonae* genes in this orthogroup identified as mdRNA candidates in this study; MOV_mdRNAs: *D. mojavensis* genes in this orthogroup identified as mdRNA candidates in this study; n_Dmel_total: total number of *D. melanogaster* genes in this orthogroup; n_Dvir_total: total number of *D. virilis* genes in this orthogroup; n_ARI_total: total number of *D. arizonae* genes in this orthogroup; n_MOV_total: total number of *D. mojavensis* genes in this orthogroup; n_mdRNA_Dmel: number of *D. melanogaster* mdRNA candidates in this orthogroup; n_mdRNA_Dvir: number of *D. virilis* mdRNA candidates in this orthogroup; n_mdRNA_ARI: number of *D. arizonae* mdRNA candidates in this orthogroup; n_mdRNA_MOV: number of *D. mojavensis* mdRNA candidates in this orthogroup.
(TXT)

S1 File Generation of the *D. arizonae* protamine-GFP sperm line. Detailed description of the molecular procedures used to generate the *D. arizonae* transgenic line expressing a Protamine-EGFP fusion protein in sperm nuclei.
(DOCX)
