# Supplementary material for: A catalog of mRNAs transferred from males to females during mating in Drosophila arizonae and D. mojavensis
Source: PLoS One. 2026 Oct 8;21(10):e0358942. doi: 10.1371/journal.pone.0358942 (PMC13648966; doi:10.1371/journal.pone.0358942)
Supplement: Supplementary file 1 [file pone.0358942.s001.pdf]

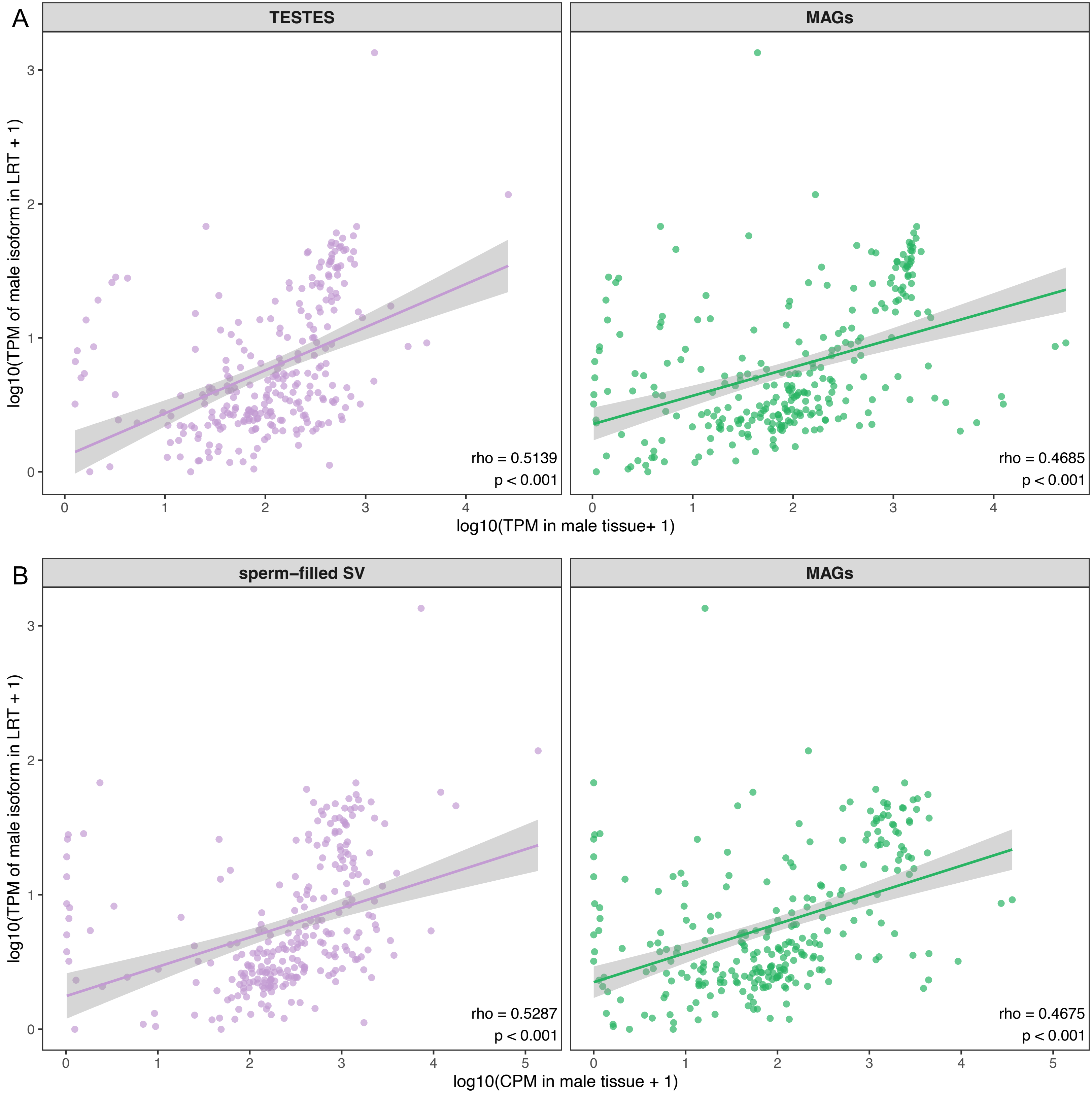
A
TESTES
MAGs
rho = 0.5139
p < 0.001
rho = 0.4685
p < 0.001
log10(TPM of male isoform in LRT + 1)
log10(TPM in male tissue+ 1)
B
sperm−filled SV
MAGs
rho = 0.5287
p < 0.001
rho = 0.4675
p < 0.001
log10(TPM of male isoform in LRT + 1)
log10(CPM in male tissue + 1)
