# Supplementary material for: A catalog of mRNAs transferred from males to females during mating in Drosophila arizonae and D. mojavensis
Source: PLoS One. 2026 Oct 8;21(10):e0358942. doi: 10.1371/journal.pone.0358942 (PMC13648966; doi:10.1371/journal.pone.0358942)
Supplement: Supplementary file 2 [file pone.0358942.s011.docx]

**Generation of the *D. arizonae* protamine-GFP sperm line**

**attP landing site insertion**

To generate a *D. arizonae* line with GFP-tagged sperm, we used plasmid insertion with an attP landing site and ϕC31 integrase (Knapp et al. 2015). The attP landing site was inserted into the genome of the ARI line using CRISPR/Cas9 with a single-stranded oligodeoxynucleotide (ssODN) donor containing the attP landing sequence. Two overlapping guide RNAs were designed using Geneious Prime (v2024; Kearse et al. 2012) targeting a location in the fifth exon of the *white* gene (ARI10968); insertion of the attP landing site into this exon disrupts the gene, generating a white-eyed fly. To produce the sgRNAs, we used a primer-dimer-based method with T7 polymerase (Bassett et al. 2013). Briefly, the DNA template was generated using NEB Q5 High Fidelity 2X Master Mix with a common reverse primer and a guide-specific forward primer. PCR was run for 35 cycles with an annealing temperature of 60°C, and the product was purified using a Qiagen QIAquick PCR Cleanup Kit. Final guide RNAs were synthesized using the NEB HiScribe T7 RNA Synthesis Kit with 16 h of incubation at 37°C, yielding a concentration of 2.4 µg/µL.

Cas9 mRNA was produced using the NEB HiScribe T7 mRNA Kit with CleanCap Reagent AG. We modified the *pCas9-polyA* plasmid (Yoshimi et al. 2016; Addgene) using the NEB Q5 Site-Directed Mutagenesis Kit to change the terminal dinucleotide of the T7 promoter from GG to AG to ensure compatibility with the CleanCap Reagent. The modified plasmid was cloned into NEB 5-alpha Competent *E. coli* (High Efficiency) cells and extracted using the ZymoPURE II Plasmid Midiprep Kit. Nanopore sequencing revealed a reduced poly(A) tail, likely due to recombination in the plasmid; to extend the tail, yeast poly(A) polymerase (Molecular Cloning Laboratories) was added following the T7 reaction. The tailed product was purified by lithium chloride precipitation and resuspended in THE RNA Storage Solution (Thermo Fisher Scientific) to a final concentration of 1 µg/µL. Embryo injection was performed on embryos less than 30 min old using a Narishige IM 300 Microinjector. The injection mix contained 5 µL of 1 µg/µL Cas9 mRNA, 1 µL of 2.4 µg/µL pooled sgRNAs, and 1 µL of 1.4 µg/µL ssODN. All primer, ssODN, and plasmid sequences are available at OSF (https://osf.io/xub8g).

Once adults eclosed, they were screened by PCR for the presence of the attP landing site using an internal site primer and an external flanking primer; positive individuals were identified by gel electrophoresis and crossed together. F1 offspring were similarly screened, and heterozygous individuals were intercrossed. In the F2 generation, primers flanking the attP site were used to identify homozygous individuals, which were intercrossed to establish a stable line. The homozygous line was verified by Sanger sequencing (Eurofins Genomics) using flanking primers to confirm correct attP site insertion.

**Protamine-GFP construct and phiC31 integration**

For the Protamine-GFP (ProtA-eGFP) construct, we followed an approach analogous to that used in *D. melanogaster*, where Protamine-GFP fusions label sperm nuclei (Manier et al. 2010). *D. melanogaster* has two Protamine paralogs, ProtA and ProtB; *D. arizonae* has a single copy, ARI17338. To rescue the *white* eye color phenotype caused by the attP site insertion, we constructed a *D. arizonae* version of the *D. melanogaster* mini-white marker gene (hereafter ARI-mini-white), designed following a similar structure to the *D. melanogaster* construct: we included 1,729 bp of upstream regulatory sequence and retained the first 409 bp and last 225 bp of intron 1, deleting 7,580 bp of intron 1, while maintaining all remaining introns and exons as well as 258 bp downstream of the stop codon. Four pieces were PCR-amplified using NEB Q5 High Fidelity 2X Master Mix to assemble the ARI-mini-white construct.

The ARI-mini-white construct was first inserted into *pBPGUw* (Pfeiffer et al. 2008; Addgene) by digesting with KpnI, NsiI, NdeI, and AatII (New England Biolabs) to remove the existing *D. melanogaster* mini-white and Gateway cloning site; the two required fragments were gel-extracted using an NEB Monarch DNA Gel Extraction Kit. A 1,582 bp regulatory region of ARI15991 was also included in the assembly as a Gal4 driver (unrelated to the present study but required by the construct design). All seven pieces were assembled using NEBuilder HiFi DNA Assembly Master Mix and cloned into NEB 5-alpha Competent *E. coli* (High Efficiency) cells to generate *pARI-Acp32CD-GAL4/ARI-minwht-attB*, which was verified by Nanopore sequencing (Plasmidsaurus). Our ARI-mini-white construct rescues red eye color and partially rescues testes pigmentation.

To generate the Protamine-GFP fusion construct, we PCR-amplified the complete coding region of ARI17338 together with 2,676 bp of upstream regulatory sequence, combined this with the EGFP coding sequence from *3xP3-EGFP-vas-dϕC31-attB* (Bischof et al. 2007) and 688 bp of the ARI17338 3′ UTR. These three pieces were assembled using NEBuilder HiFi DNA Assembly Master Mix into *pARI-Acp32CD-GAL4/ARI-minwht-attB* linearized with NotI and HindIII, generating *pARI-ProtA-EGFP/ARI-minwht-attB*. After Nanopore verification, the plasmid was cloned into NEB 5-alpha Competent *E. coli* (High Efficiency) cells, extracted using the ZymoPURE II Plasmid Midiprep Kit, further purified by sodium acetate–ethanol precipitation, and resuspended in water to a concentration of 2.4 µg/µL.

ϕC31 integrase mRNA was produced by modifying the CleanCap Cas9 mRNA plasmid described above, replacing the Cas9 coding sequence with the ϕC31 integrase sequence from *3xP3-EGFP-vas-dϕC31-attB* (Bischof et al. 2007). The *cas9* sequence was removed by inverse PCR using NEB Q5 High Fidelity 2X Master Mix, the integrase fragment was PCR-amplified from the same template, and the two pieces were joined using NEBuilder HiFi DNA Assembly Master Mix; the sequence was verified by Nanopore sequencing. mRNA synthesis followed the same protocol as for Cas9 mRNA. Embryo injection was performed as described above, with an injection cocktail containing 5 µL of 1 µg/µL ϕC31 integrase mRNA and 1 µL of 2.4 µg/µL *pARI-ProtA-EGFP/ARI-minwht-attB*. All eclosed adults were crossed to white-eyed *attP* landing site virgins, and F1 offspring were screened for rescue of red eye color. Positive F1 individuals were intercrossed, and F2 offspring were assessed for red eye intensity, with homozygous individuals identified by more intense pigmentation. GFP expression in sperm was visually confirmed in transformed males (*attP*{*ARI-ProtA-eGFP/ARI-minwht*} *w*^CRISPR^).

**References**

Bassett, A.R., Tibbit, C., Ponting, C.P. & Liu, J.L. (2013) Highly efficient targeted mutagenesis of Drosophila with the CRISPR/Cas9 system. Cell Reports, 4, 220–228. Available from: https://doi.org/10.1016/j.celrep.2013.06.020

Bischof, J., Maeda, R.K., Hediger, M., Karch, F. & Basler, K. (2007) An optimized transgenesis system for Drosophila using germ-line-specific ϕC31 integrases. Proceedings of the National Academy of Sciences USA, 104, 3312–3317. Available from: https://doi.org/10.1073/pnas.0611511104

Kearse, M., Moir, R., Wilson, A., Stones-Havas, S., Cheung, M., Sturrock, S. et al. (2012) Geneious Basic: an integrated and extendable desktop software platform for the organization and analysis of sequence data. Bioinformatics, 28, 1647–1649. Available from: https://doi.org/10.1093/bioinformatics/bts199

Knapp, J.M., Chung, P. & Simpson, J.H. (2015) Generating customized transgene landing sites and multi-transgene arrays in Drosophila using phiC31 integrase. Genetics, 199, 919–934. Available from: https://doi.org/10.1534/genetics.114.173187

Manier, M.K., Bauer, J.M., Lüpold, S., Belote, J.M., Scott, W., Starmer, W.T. et al. (2010) Resolving mechanisms of competitive fertilization success in Drosophila melanogaster. Science, 328, 354–357. Available from: https://doi.org/10.1126/science.1187096

Pfeiffer, B.D., Jenett, A., Hammonds, A.S., Ngo, T.T., Misra, S., Murphy, C. et al. (2008) Tools for neuroanatomy and neurogenetics in Drosophila. Proceedings of the National Academy of Sciences USA, 105, 9715–9720. Available from: https://doi.org/10.1073/pnas.0803697105

Ramírez, F., Ryan, D.P., Grüning, B., Bhardwaj, V., Kilpert, F., Richter, A.S. et al. (2016) deepTools2: a next generation web server for deep-sequencing data analysis. Nucleic Acids Research, 44, W160–W165. Available from: https://doi.org/10.1093/nar/gkw257

Yoshimi, K., Kunihiro, Y., Kaneko, T., Nagahora, H., Voigt, B. & Mashimo, T. (2016) ssODN-mediated knock-in with CRISPR-Cas for large genomic regions in zygotes. Nature Communications, 7, 10431. Available from: https://doi.org/10.1038/ncomms10431

**Supplementary file S1. Generation of the *D. arizonae* protamine-GFP sperm line**

**attP landing site insertion**

To generate a *D. arizonae* line with GFP-tagged sperm, we used plasmid insertion with an attP landing site and ϕC31 integrase (Knapp et al. 2015). The attP landing site was inserted into the genome of the ARI line using CRISPR/Cas9 with a single-stranded oligodeoxynucleotide (ssODN) donor containing the attP landing sequence. Two overlapping guide RNAs were designed using Geneious Prime (v2024; Kearse et al. 2012) targeting a location in the fifth exon of the *white* gene (ARI10968); insertion of the attP landing site into this exon disrupts the gene, generating a white-eyed fly. To produce the sgRNAs, we used a primer-dimer-based method with T7 polymerase (Bassett et al. 2013). Briefly, the DNA template was generated using NEB Q5 High Fidelity 2X Master Mix with a common reverse primer and a guide-specific forward primer. PCR was run for 35 cycles with an annealing temperature of 60°C, and the product was purified using a Qiagen QIAquick PCR Cleanup Kit. Final guide RNAs were synthesized using the NEB HiScribe T7 RNA Synthesis Kit with 16 h of incubation at 37°C, yielding a concentration of 2.4 µg/µL.

Cas9 mRNA was produced using the NEB HiScribe T7 mRNA Kit with CleanCap Reagent AG. We modified the *pCas9-polyA* plasmid (Yoshimi et al. 2016; Addgene) using the NEB Q5 Site-Directed Mutagenesis Kit to change the terminal dinucleotide of the T7 promoter from GG to AG to ensure compatibility with the CleanCap Reagent. The modified plasmid was cloned into NEB 5-alpha Competent *E. coli* (High Efficiency) cells and extracted using the ZymoPURE II Plasmid Midiprep Kit. Nanopore sequencing revealed a reduced poly(A) tail, likely due to recombination in the plasmid; to extend the tail, yeast poly(A) polymerase (Molecular Cloning Laboratories) was added following the T7 reaction. The tailed product was purified by lithium chloride precipitation and resuspended in THE RNA Storage Solution (Thermo Fisher Scientific) to a final concentration of 1 µg/µL. Embryo injection was performed on embryos less than 30 min old using a Narishige IM 300 Microinjector. The injection mix contained 5 µL of 1 µg/µL Cas9 mRNA, 1 µL of 2.4 µg/µL pooled sgRNAs, and 1 µL of 1.4 µg/µL ssODN. All primer, ssODN, and plasmid sequences are provided in the supplementary files (XXXOSF linkXXX).

Once adults eclosed, they were screened by PCR for the presence of the attP landing site using an internal site primer and an external flanking primer; positive individuals were identified by gel electrophoresis and crossed together. F1 offspring were similarly screened, and heterozygous individuals were intercrossed. In the F2 generation, primers flanking the attP site were used to identify homozygous individuals, which were intercrossed to establish a stable line. The homozygous line was verified by Sanger sequencing (Eurofins Genomics) using flanking primers to confirm correct attP site insertion.

**Protamine-GFP construct and phiC31 integration**

For the Protamine-GFP (ProtA-eGFP) construct, we followed an approach analogous to that used in *D. melanogaster*, where Protamine-GFP fusions label sperm nuclei (Manier et al. 2010). *D. melanogaster* has two Protamine paralogs, ProtA and ProtB; *D. arizonae* has a single copy, ARI17338. To rescue the *white* eye color phenotype caused by the attP site insertion, we constructed a *D. arizonae* version of the *D. melanogaster* mini-white marker gene (hereafter ARI-mini-white), designed following a similar structure to the *D. melanogaster* construct: we included 1,729 bp of upstream regulatory sequence and retained the first 409 bp and last 225 bp of intron 1, deleting 7,580 bp of intron 1, while maintaining all remaining introns and exons as well as 258 bp downstream of the stop codon. Four pieces were PCR-amplified using NEB Q5 High Fidelity 2X Master Mix to assemble the ARI-mini-white construct.

The ARI-mini-white construct was first inserted into *pBPGUw* (Pfeiffer et al. 2008; Addgene) by digesting with KpnI, NsiI, NdeI, and AatII (New England Biolabs) to remove the existing *D. melanogaster* mini-white and Gateway cloning site; the two required fragments were gel-extracted using an NEB Monarch DNA Gel Extraction Kit. A 1,582 bp regulatory region of ARI15991 was also included in the assembly as a Gal4 driver (unrelated to the present study but required by the construct design). All seven pieces were assembled using NEBuilder HiFi DNA Assembly Master Mix and cloned into NEB 5-alpha Competent *E. coli* (High Efficiency) cells to generate *pARI-Acp32CD-GAL4/ARI-minwht-attB*, which was verified by Nanopore sequencing (Plasmidsaurus). Our ARI-mini-white construct rescues red eye color and partially rescues testes pigmentation.

To generate the Protamine-GFP fusion construct, we PCR-amplified the complete coding region of ARI17338 together with 2,676 bp of upstream regulatory sequence, combined this with the EGFP coding sequence from *3xP3-EGFP-vas-dϕC31-attB* (Bischof et al. 2007) and 688 bp of the ARI17338 3′ UTR. These three pieces were assembled using NEBuilder HiFi DNA Assembly Master Mix into *pARI-Acp32CD-GAL4/ARI-minwht-attB* linearized with NotI and HindIII, generating *pARI-ProtA-EGFP/ARI-minwht-attB*. After Nanopore verification, the plasmid was cloned into NEB 5-alpha Competent *E. coli* (High Efficiency) cells, extracted using the ZymoPURE II Plasmid Midiprep Kit, further purified by sodium acetate–ethanol precipitation, and resuspended in water to a concentration of 2.4 µg/µL.

ϕC31 integrase mRNA was produced by modifying the CleanCap Cas9 mRNA plasmid described above, replacing the Cas9 coding sequence with the ϕC31 integrase sequence from *3xP3-EGFP-vas-dϕC31-attB* (Bischof et al. 2007). The *cas9* sequence was removed by inverse PCR using NEB Q5 High Fidelity 2X Master Mix, the integrase fragment was PCR-amplified from the same template, and the two pieces were joined using NEBuilder HiFi DNA Assembly Master Mix; the sequence was verified by Nanopore sequencing. mRNA synthesis followed the same protocol as for Cas9 mRNA. Embryo injection was performed as described above, with an injection cocktail containing 5 µL of 1 µg/µL ϕC31 integrase mRNA and 1 µL of 2.4 µg/µL *pARI-ProtA-EGFP/ARI-minwht-attB*. All eclosed adults were crossed to white-eyed *attP* landing site virgins, and F1 offspring were screened for rescue of red eye color. Positive F1 individuals were intercrossed, and F2 offspring were assessed for red eye intensity, with homozygous individuals identified by more intense pigmentation. GFP expression in sperm was visually confirmed in transformed males (*attP*{*ARI-ProtA-eGFP/ARI-minwht*} *w*^CRISPR^).

**REFERENCES**

Bassett, A.R., Tibbit, C., Ponting, C.P. & Liu, J.L. (2013) Highly efficient targeted mutagenesis of Drosophila with the CRISPR/Cas9 system. Cell Reports, 4, 220–228. Available from: https://doi.org/10.1016/j.celrep.2013.06.020

Bischof, J., Maeda, R.K., Hediger, M., Karch, F. & Basler, K. (2007) An optimized transgenesis system for Drosophila using germ-line-specific ϕC31 integrases. Proceedings of the National Academy of Sciences USA, 104, 3312–3317. Available from: https://doi.org/10.1073/pnas.0611511104

Kearse, M., Moir, R., Wilson, A., Stones-Havas, S., Cheung, M., Sturrock, S. et al. (2012) Geneious Basic: an integrated and extendable desktop software platform for the organization and analysis of sequence data. Bioinformatics, 28, 1647–1649. Available from: https://doi.org/10.1093/bioinformatics/bts199

Knapp, J.M., Chung, P. & Simpson, J.H. (2015) Generating customized transgene landing sites and multi-transgene arrays in Drosophila using phiC31 integrase. Genetics, 199, 919–934. Available from: https://doi.org/10.1534/genetics.114.173187

Manier, M.K., Bauer, J.M., Lüpold, S., Belote, J.M., Scott, W., Starmer, W.T. et al. (2010) Resolving mechanisms of competitive fertilization success in Drosophila melanogaster. Science, 328, 354–357. Available from: https://doi.org/10.1126/science.1187096

Pfeiffer, B.D., Jenett, A., Hammonds, A.S., Ngo, T.T., Misra, S., Murphy, C. et al. (2008) Tools for neuroanatomy and neurogenetics in Drosophila. Proceedings of the National Academy of Sciences USA, 105, 9715–9720. Available from: https://doi.org/10.1073/pnas.0803697105

Ramírez, F., Ryan, D.P., Grüning, B., Bhardwaj, V., Kilpert, F., Richter, A.S. et al. (2016) deepTools2: a next generation web server for deep-sequencing data analysis. Nucleic Acids Research, 44, W160–W165. Available from: https://doi.org/10.1093/nar/gkw257

Yoshimi, K., Kunihiro, Y., Kaneko, T., Nagahora, H., Voigt, B. & Mashimo, T. (2016) ssODN-mediated knock-in with CRISPR-Cas for large genomic regions in zygotes. Nature Communications, 7, 10431. Available from: https://doi.org/10.1038/ncomms10431
