# Supplementary material for: A catalog of mRNAs transferred from males to females during mating in Drosophila arizonae and D. mojavensis
Source: PLoS One. 2026 Oct 8;21(10):e0358942. doi: 10.1371/journal.pone.0358942 (PMC13648966; doi:10.1371/journal.pone.0358942)
Supplement: Peer review history

# Peer review: Decision Letter 0

**DOI:** 10.1371/journal.pone.0358942.r001

**Authors:** Joël R Drevet

8 Sep 2026

A catalog of mRNAs transferred from males to females during mating in *Drosophila arizonae*  and *D. mojavensis*

PONE-D-26-35725

Dear Dr.  Hurtado,

We’re pleased to inform you that your manuscript has been judged scientifically suitable for publication and will be formally accepted for publication once it meets all outstanding technical requirements.

Within one week, you’ll receive an e-mail detailing the required amendments. When these have been addressed, you’ll receive a formal acceptance letter and your manuscript will be scheduled for publication.

An invoice will be generated when your article is formally accepted. Please note, if your institution has a publishing partnership with PLOS and your article meets the relevant criteria, all or part of your publication costs will be covered. Please make sure your user information is up-to-date by logging into Editorial Manager at Editorial Manager® and clicking the ‘Update My Information' link at the top of the page. For questions related to billing, please contact billing support.

If your institution or institutions have a press office, please notify them about your upcoming paper to help maximize its impact. If they’ll be preparing press materials, please inform our press team as soon as possible -- no later than 48 hours after receiving the formal acceptance. Your manuscript will remain under strict press embargo until 2 pm Eastern Time on the date of publication. For more information, please contact onepress@plos.org.

Kind regards,

Prof. Joël R Drevet, Ph.D.

Academic Editor

PLOS One

Journal Requirements:

When submitting your revision, we need you to address these additional requirements.

1.Please ensure that your manuscript meets PLOS ONE's style requirements, including those for file naming. The PLOS ONE style templates can be found at

https://journals.plos.org/plosone/s/file?id=wjVg/PLOSOne_formatting_sample_main_body.pdf and

https://journals.plos.org/plosone/s/file?id=ba62/PLOSOne_formatting_sample_title_authors_affiliations.pdf

Additional Editor Comments:

This is a well-done piece of work that doesn't need any changes at this stage. Good job, well done!

Reviewers' comments:

Reviewer's Responses to Questions

**Comments to the Author**

1. Is the manuscript technically sound, and do the data support the conclusions?

Reviewer #1: Yes

Reviewer #2: Yes

**********

2. Has the statistical analysis been performed appropriately and rigorously?

Reviewer #1: Yes

Reviewer #2: Yes

**********

3. Have the authors made all data underlying the findings in their manuscript fully available?

The PLOS Data policy

Reviewer #1: Yes

Reviewer #2: Yes

**********

4. Is the manuscript presented in an intelligible fashion and written in standard English?

Reviewer #1: Yes

Reviewer #2: Yes

**********

Reviewer #1: General Assessment:

This is an outstanding and methodologically rigorous study that substantially advances our understanding of intercellular RNA transfer during reproduction. The authors have systematically characterized the repertoire of male-derived mRNAs (mdRNAs) transferred to females during mating in two cactophilic Drosophila species, building upon their previous landmark discovery that these transcripts can be translated into functional proteins in the female reproductive tract.

The work is comprehensive, technically sound, and provides a high-confidence catalog of 270 mdRNA candidates. The integration of transcriptomic (both whole-transcript and 3′-end RNA-seq), proteomic, and live-imaging approaches is particularly impressive and lends strong support to their conclusions regarding tissue origins and the potential mechanisms of RNA release.

Significance and Broader Impact:

The significance of this work extends well beyond basic evolutionary biology. The catalog and mechanisms revealed here have the potential to serve as powerful tools for epigenetic research, providing new insights into how paternal transcripts can influence offspring development and reproductive success. Furthermore, the principles uncovered in this Drosophila model could have translational applications in genetic research, animal and plant breeding improvement, and potentially inform our understanding of human reproductive biology and infertility. This study therefore has both fundamental and applied implications that make it of broad interest to the scientific community.

Methods and Data Quality:

The methodological approach is a clear strength. The four-filter pipeline is appropriately conservative, prioritizing specificity to minimize false positives. The inclusion of multiple controls (conspacific crosses, head tissue) demonstrates excellent experimental design. The multi-omics integration provides a robust and multi-dimensional characterization of mdRNA origins, and the GFP-labeled sperm tracking experiment offers a novel and plausible mechanistic hypothesis for RNA release from trapped sperm cells.

Clarity and Presentation:

The manuscript is exceptionally well-written, logically organized, and clearly presented. The figures are informative and effectively convey the key findings. The discussion thoughtfully contextualizes the results within the broader literature while honestly acknowledging the study's limitations.

Recommendation:

Accept. This work is scientifically rigorous, presents novel and important findings, and is of broad interest to the readership of PLOS ONE. The study is complete as presented, and I enthusiastically recommend its publication without any further revisions.

Reviewer #2: This is a nicely experimentally executed and written study. Overall it provides a useful resource for the study of mdRNAs in flies. Further it introduces and interesting potential mechanism for how mdRNAs are released by sperm and then accessed by the female reproductive tract. Any comments I have would be very minor. I recommend publication.

**********

what does this mean?). If published, this will include your full peer review and any attached files.

If you choose “no”, your identity will remain anonymous but your review may still be made public.

**Do you want your identity to be public for this peer review?**  For information about this choice, including consent withdrawal, please see our Privacy Policy

Reviewer #1: **Yes:**  Weiming Chen

Reviewer #2: No

**********

# Editor report: Acceptance letter

**DOI:** 10.1371/journal.pone.0358942.r002

**Authors:** Joël R Drevet

PONE-D-26-35725

PLOS One

Dear Dr. Hurtado,

I'm pleased to inform you that your manuscript has been deemed suitable for publication in PLOS One. Congratulations! Your manuscript is now being handed over to our production team.

At this stage, our production department will prepare your paper for publication. This includes ensuring the following:

* All references, tables, and figures are properly cited

* All relevant supporting information is included in the manuscript submission,

* There are no issues that prevent the paper from being properly typeset

You will receive further instructions from the production team, including instructions on how to review your proof when it is ready. Please keep in mind that we are working through a large volume of accepted articles, so please give us a few days to review your paper and let you know the next and final steps.

Lastly, if your institution or institutions have a press office, please let them know about your upcoming paper now to help maximize its impact. If they'll be preparing press materials, please inform our press team within the next 48 hours. Your manuscript will remain under strict press embargo until 2 pm Eastern Time on the date of publication. For more information, please contact onepress@plos.org.

You will receive an invoice from PLOS for your publication fee after your manuscript has reached the completed accept phase. If you receive an email requesting payment before acceptance or for any other service, this may be a phishing scheme. Learn how to identify phishing emails and protect your accounts at https://explore.plos.org/phishing.

If we can help with anything else, please email us at customercare@plos.org.

Thank you for submitting your work to PLOS One and supporting open access.

Kind regards,

PLOS One Editorial Office Staff

on behalf of

Prof. Joël R Drevet

Academic Editor

PLOS One
